# SV-AUTOPILOT: optimized, automated construction of structural variation discovery and benchmarking pipelines

**DOI:** 10.1186/s12864-015-1376-9

**Published:** 2015-03-25

**Authors:** Wai Yi Leung, Tobias Marschall, Yogesh Paudel, Laurent Falquet, Hailiang Mei, Alexander Schönhuth, Tiffanie Yael Maoz (Moss)

**Affiliations:** Sequencing Analysis Support Core, Leiden University Medical Center, Leiden, The Netherlands; Center for Bioinformatics, Saarland University, Saarbrücken, Germany; Max Planck Institute for Informatics, Saarbrücken, Germany; Centrum Wiskunde and Informatica, Amsterdam, The Netherlands; Animal Breeding and Genomics Centre, Wageningen University, Wageningen, The Netherlands; University of Fribourg and Swiss Institute of Bioinformatics, Fribourg, Switzerland; Weizmann Institute of Science, Rehovot, Israel

**Keywords:** Structural Variation, SV tool, Meta-tool, Non-human genome, Standardized pipeline, SV prediction, Benchmarking, Next-Generation Sequencing Analysis, SV tool development

## Abstract

**Background:**

Many tools exist to predict structural variants (SVs), utilizing a variety of algorithms. However, they have largely been developed and tested on human germline or somatic (e.g. cancer) variation. It seems appropriate to exploit this wealth of technology available for humans also for other species. Objectives of this work included:

- Creating an automated, standardized pipeline for SV prediction.
- Identifying the best tool(s) for SV prediction through benchmarking.
- Providing a statistically sound method for merging SV calls.

**Results:**

The SV-AUTOPILOT meta-tool platform is an automated pipeline for standardization of SV prediction and SV tool development in paired-end next-generation sequencing (NGS) analysis. SV-AUTOPILOT comes in the form of a virtual machine, which includes all datasets, tools and algorithms presented here. The virtual machine easily allows one to add, replace and update genomes, SV callers and post-processing routines and therefore provides an easy, out-of-the-box environment for complex SV discovery tasks. SV-AUTOPILOT was used to make a direct comparison between 7 popular SV tools on the *Arabidopsis thaliana* genome using the Landsberg (Ler) ecotype as a standardized dataset. Recall and precision measurements suggest that Pindel and Clever were the most adaptable to this dataset across all size ranges while Delly performed well for SVs larger than 250 nucleotides. A novel, statistically-sound merging process, which can control the false discovery rate, reduced the false positive rate on the Arabidopsis benchmark dataset used here by >60%.

**Conclusion:**

SV-AUTOPILOT provides a meta-tool platform for future SV tool development and the benchmarking of tools on other genomes using a standardized pipeline. It optimizes detection of SVs in non-human genomes using statistically robust merging. The benchmarking in this study has demonstrated the power of 7 different SV tools for analyzing different size classes and types of structural variants. The optional merge feature enriches the call set and reduces false positives providing added benefit to researchers planning to validate SVs. SV-AUTOPILOT is a powerful, new meta-tool for biologists as well as SV tool developers.

**Electronic supplementary material:**

The online version of this article (doi:10.1186/s12864-015-1376-9) contains supplementary material, which is available to authorized users.

## Background

Structural variations (SVs) are the main source of intra- and interspecies variation and have been shown to play an important role in the evolution of many species [1-4]. SV detection is now playing a leading role in the advancement of research for many organisms such as plant breeding and our understanding of human diseases and disorders [5,6]. Indeed, SVs are of interest to researchers from varying backgrounds aiming to address SVs from different angles. Therefore the need to identify the most efficient and reliable tools for SV analysis is critical to the advancement of genomic research for all organisms.

Large genomic structural variants, such as insertions and deletions of more than 20 base pairs (bp), copy number variants and translocations are often induced during the course of DNA repair. Several DNA repair mechanisms exist in plants and animals, but usage may vary according to structure and arrangement of the genome being studied. Some relevant, SV-inducing mechanisms include non-homologous end-joining (NHEJ) associated with DNA-repair at regions with very limited or no homology, non-allelic homologous recombination (NAHR) in highly similar regions (unequal cross-over), fork stalling and template switching (FoSTeS) as in replication-error mechanisms, and finally transposable element (TE)-mediated mechanisms of repair [see [7] for a more detailed review of genomic technologies and computational techniques currently used to measure SVs].

In addition to variations in the types of SV induced, genomes may also vary in their degree of complexity. In contrast to vertebrate genomes, for example, plant genomes are more susceptible to hybridization and to further increases of genome complexity [8]. These challenges can often exacerbate the numbers of sequencing errors and mapping uncertainties which further add to the complexity of identifying structural variants [9]. This may lead to differences in behavior of the SV detection tools that were solely designed with *Homo sapiens* or animal data in mind. While previous studies have sought to address problems of sequencing errors and mapping uncertainties in human genomes with the development of new SV tools [10,11], we are motivated by the need for insight into the performance of SV tools on non-human genomes. It is critical that multiple tools be used in identifying SVs as each tool is likely to respond to these changes in genome structure with varying degrees of success [12]. This should be taken into consideration when choosing a SV detection tool(s) as some are more suited to one purpose than another. For this reason we have chosen to benchmark tools using varying SV techniques.

### SV detection techniques

Four general techniques are employed to detect structural variations from paired-end sequencing data. Each approach has merits and shortcomings. Here we provide a brief sketch of each technique and list a few tools which make use of them.

**Coverage**: The coverage, that is the amount of reads aligning to a genomic region, can be used to draw conclusions on its copy number status. When a region is not covered by any reads, for instance, one can conclude that the respective part is not present in the genome under investigation. An advantage of this technique is that it allows for a direct estimate of the copy number. However this technique only applies to larger events and can be affected by sequencing biases. In general, this type of methods works best for comparing pairs of samples sequenced using the same platform/protocol. Examples of such tools include CNVer and CNVnator [13,14].

**Internal segment size (paired-end reads and mate-pairs)**: The internal segment (IS) is the unsequenced part between the two read ends in a paired-end sequenced (genomic) fragment. Library preparation and sequencing protocols determine the shape of the distribution of internal segment sizes. When alignments at a particular locus give rise to estimates of an IS size that deviates significantly from this background distribution, the locus is likely to be affected by a structural variation in the genome being examined. As tools draw conclusions based on statistics of IS length, their performance rates crucially depend on the shape of those distributions. In general, they perform best for unimodal distributions with a small standard deviation. As the observed IS size increases in the presence of insertions, the maximal length of insertions that can be detected is limited by the mean IS size. This limitation, however, does not exist for deletions. Examples of IS size-based SV discovery tools include Breakdancer, CLEVER, GASV, HYDRA, Modil, SVDetect and VariationHunter [10,11,15-19].

**Split-reads:** Split-read methods try to align reads across structural variation breakpoints. That is, one of the two read ends is aligned such that the SV is part of the unaligned read. This technique has the advantage of yielding single base pair resolution. However, performance is dependent on the length of the reads as shorter reads lead to more, ambiguous (split-read) alignments, especially in repetitive regions of the genome. Examples of such tools include PINDEL, SplazerS, and CREST [20-22]. Standard read mappers like BWA, Bowtie, GSNAP or Stampy, to a certain extent, can also provide correct, gapped alignments for insertions and deletions (indels) shorter than 50 bp [23-26].

**Local assembly**: Structural variations can also be detected by running a *de novo* assembly and comparing the resulting contigs with the reference genome. This method is unbiased, yields single base-pair resolution and is, in general, the only way of detecting insertions of novel sequence longer than the read length. Short reads and repetitive areas, however, make it difficult to build sufficiently long contigs from NGS reads. Examples of assembly tools include ALLPATHS, SOAPdenovo and VELVET [27-29].

**Combined:** In recent years, several hybrid methods using more than one of these four paradigms have been developed (e.g. DELLY, MATE-CLEVER, PRISM, and SV-seq2 [30-33]).

### Creating a “Meta-Tool” for All Organisms

The difficulty in selecting tools for SV prediction in non-human genomes is manifold.

- First, tools developed so far are often tailored towards human or vertebrate genomes [11,19,20,30-32]. That is, tools may expect genomes to be diploid and of a certain repetitive structure, gene count, CG content and so on. Without further analysis, it remains unclear which tools are robust with respect to changes in GC content, complexity and, last but not least, ploidy. Reliable benchmark datasets that reflect such modifications are required to properly evaluate tool performance.
- Second**,** creating an optimal selection of SV calls from the applicable tools is another, involved issue. To do this, one needs a statistically sound procedure by which to create reliable and strong consensus call sets from the tools chosen, and one, again, needs to rigorously evaluate such consensus call sets.
- Third, tools should be evaluated in a standardized pipeline. Because SV discovery is still a relatively recent and active area of research, benchmark datasets that reflect both true sequence context and SV abundance are hardly available [12]. What confounds all the issues further is that many tools, due to being in active use, frequently undergo updates, which may decisively touch upon their strengths and weaknesses.

Testing and comparing multiple tools in a standardized fashion is daunting for both researchers and programmers. A “meta-tool” platform addresses these many considerations as it is flexible with respect to frequent version updates, integration of new tools and new datasets. Here, we provide such a platform in the SV-AUTOPILOT Virtual Machine. This platform allows for

- Evaluation of new tools.
- Running multiple SV callers in a single run from an easy, out-of-the-box program.
- Interoperability with downstream analysis as all outputs are in VCF format.

In order to identify the SV tool(s) most adaptable to non-human genomic research, we further propose to benchmark all tools of interest on known genomes with validated SVs through a standardized pipeline.

In summary, we present SV-AUTOPILOT, a Structural Variation AUTOmated PIpeLine Optimization Tool. SV-AUTOPILOT standardizes the SV detection pipeline and can be used on existing computing infrastructure in the form of a Virtual Machine (VM) Image. Modularization of components allows for easy integration of additional tools, version updates and other benchmark datasets. In addition, the benchmarking data of tool performance and computational demands provided here demonstrates the critical need for using multiple SV tools for predicting SVs. Using this platform, researchers are able to identify SVs from multiple SV detection tools with the choice of merging the call sets according to the statistically-sound approach provided here. False positives are thereby reduced and the call set becomes enriched for ‘true’ SV events. SV-AUTOPILOT provides a much needed resource for biomedical researchers, bioinformaticians and tool developers. The SV-AUTOPILOT is available with a user guide via the open source repository GitHub https://github.com/ALLBio/allbiotc2 and the VM is hosted on the ALLBIO web site https://bioimg.org/sv-autopilot.

## Methods

### Benchmarking: datasets

Tools were benchmarked on a reconstructed genome using validated SVs from the *Arabidopsis thaliana* Lansberg (Ler) ecotype [34]. The SV calls were incorporated into the TAIR9 genomic sequence as per the procedure previously described [15] for Craig Venter’s genome [35]. Reads were simulated to correspond to Illumina HiSeq paired-end 100 bp read data with a fragment size of 500 bp and 30x coverage, using simseq with the Illumina HiSeq error profile [36].

Most SV tools were developed for human or animal genomes [11,19,20,30-32]. In order to compare the performance of the tools on human and plant genomes, simulated reads from the human chromosome 21 of Venter’s genome were used (see [15,35]), where read simulation proceeded analogously to the read simulation for the ‘Ler’ Arabidopsis genome. In this way, reads correspond to Illumina HiSeq paired-end 100 bp read data with a fragment size of 500 bp and 30x coverage. We chose Venter’s genome as the set of variants arising from it make an independent, high-quality choice of a set of variants which has already been used in previous studies [15,16,31]. Most importantly, this set of “truth” variants does not suffer from tool-specific biases, as, for example, sets of variants obtained from the 1000 Genomes project. As the variants from the 1000 Genomes project stem from computational tools, those tools would clearly outperform the others when evaluated on such datasets.

We determined two standard deviation (sd) settings for insert sizes, one of which reflects a popular, realistic scenario (sd = 15, [37]) and the other one of which represents a “worst-case” scenario (sd = 50), which reflects less optimal sequencing library protocols. This analysis highlights how the performance rates of tools behave relative to increased standard deviation. Although we can expect much better sd values using the latest technologies, an sd of 50 is not atypical. The 1000 Genomes project, for instance, contains samples with sd values in this range. Table 1 provides detailed parameters for each of the datasets used here. Note that, one can “downsample” these datasets to also emulate scenarios of lower coverage if desired.

**Table 1:** **Overview of test datasets**

**Type**	**Genome**	**Sequencer**	**Length(bp)**	**Insertsize (bp)**	**Insert sd**	**Coverage**
**Illumina 1.9 FastQ Paired End**	**Tair9**	**SimSeq Illumina Profile**	**100**	**500**	**15**	**30x**
**Illumina 1.9 FastQ Paired End**	**Tair9**	**SimSeq Illumina Profile**	**100**	**500**	**50**	**30x**
Illumina 1.9 FastQ Paired End	Human Genome hg19	SimSeq Illumina Profile	100	500	15	30x
Illumina 1.9 FastQ Paired End	Human Genome hg19	SimSeq Illumina Profile	100	500	50	30x

Arabisopsis (Tair9) and Human (hg19) datasets were simulated using a SimSeq Illumina 1.9 Paired End profile with 100 bp reads and an insert size of 500. Two standard deviations of insert size were created for each dataset, more ideal (15) and less ideal (50). All datasets were simulated to 30x coverage.

### Benchmarking: SV tools

For benchmarking, several well-known SV discovery tools were selected as defined by the following criteria:

- Open source (for the sake of comparing algorithms).
- Support of command line mode (excludes tools requiring a graphical user interface).
- Default parameters provided and applicable in all cases considered here.
- Scaled to process a moderate size genome with the operating limits of a common laptop.

The tools selected varied in terms of their approaches. We included paired-end methods, split-read methods and combinations thereof, as those combined approaches reflect the state-of-the-art in indel discovery. In detail, we selected Breakdancer, Clever, Delly, GASV, Pindel, Prism, and SVDetect [11,15,16,19,20,30] for being included in SV-AUTOPILOT, as a selection of well-known state-of-the-art SV discovery tools. In addition, the modularized structure of SV-AUTOPILOT conveniently allows one to replace and add tools according to individual preferences. All tools were run using the most recent releases available as of January 30, 2014. Although many tools are able to predict varied types of structural variants (e.g. also inversions, translocations and mixed events beyond insertions and deletions), the emphasis here is on insertions and deletions of more than 20 bp. We leave an extension of our platform towards those other classes of SVs as promising future work. All technology presented here is easily adapted to also allow for these extensions.

Discovery of insertions and deletions smaller than 20 bp is a prevalent part of variant discovery pipelines (GATK, [38]) and poses no further challenges to the user, while discovery of indels greater than 20 bp still comes with substantial difficulties. Therefore, the focus of this work will be on insertions and deletions larger than 20 bp. They may be referred to as ‘indels’ or structural variants (SVs) although we recognize that this may occasionally clash with existing nomenclature.

### Benchmarking: SV size classes

For the purposes of benchmarking, insertions and deletions events were divided into 5 size categories: 20-49 bp, 50-99 bp, 100-249 bp, 250-999 bp, 1kbp-50kbp. Distinguishing between those size classes allows one to identify size-dependent strengths and weaknesses of the tools considered. The first class, 20-49 bp is, to a certain degree, still in reach of even ordinary alignment tools, while generally constituting the major area of activity of split-read aligners. 50-99 bp can in general be considered as the most difficult size range, where both split-read aligners and IS based approaches face non-negligible challenges. Overall, the first three size ranges, 20–49 bp, 50–99 bp and 100-249 bp have sometimes been referred to as the twilight zone of SV calls as all of them are rather difficult to identify. Above 250 bp, the SVs are usually larger than the insert range, which makes calling them relatively easy for IS based approaches. We determined 50Kb, the size of the largest validated SV documented in test datasets, as an upper limit for the purposes of the benchmarking documentation provided here. It is noteworthy that most tools that are able to detect 50Kb SVs can also detect larger SVs. Validated SV counts in the various size classes for both (Human and Arabidopsis) data sets are provided in Table 2.

**Table 2:** **Counts of Validated SVs used to benchmark SV tool performance**

**Data type**	**Type**	**Length 20-49**	**Length 50-99**	**Length 100-249**	**Length 250-999**	**Length 1000-50000**
Human chr. 21	insertion	136	37	30	19	10
**Tair v.9**	**insertion**	**8094**	**446**	**82**	**44**	**3**
Human chr. 21	*deletion*	118	33	19	19	4
**Tair v.9**	***deletion***	**3595**	**781**	**393**	**572**	**370**

### Virtual machine

Virtual machines (VM) ensure the consistency, reproducibility and reliability of our test environment. Each VM was equipped with the same software installation. The virtual machines were installed with Ubuntu 12.04.3 LTS using default configuration. After installation of the essential system components, the software of each SV tool was installed, and a non-persistent system image was cloned from the master machine. The non-persistent image provides a consistent and reliable working environment to run the benchmarking analyses.

For initial testing of computational performance by each tool, VMs were created for varying numbers of CPU cores (4/8/12/16/32) and varying amounts of available main memory (32/64/96/128/256 GB). Multiple machines were booted with this non-persistent image, SV discovery tools were run and the resulting data was collected. Different settings were tested to explore the computational resource requirements of the SV discovery tools.

### Analysis pipeline

SV-AUTOPILOT was implemented using Makefiles according to the GNU Make syntax. A modularized setup was employed, which allows one to disable and replace aligners and SV discovery tools as needed or as per personal preference. Additionally, such modularity enables tool-wise parallelization.

**Figure 1:**
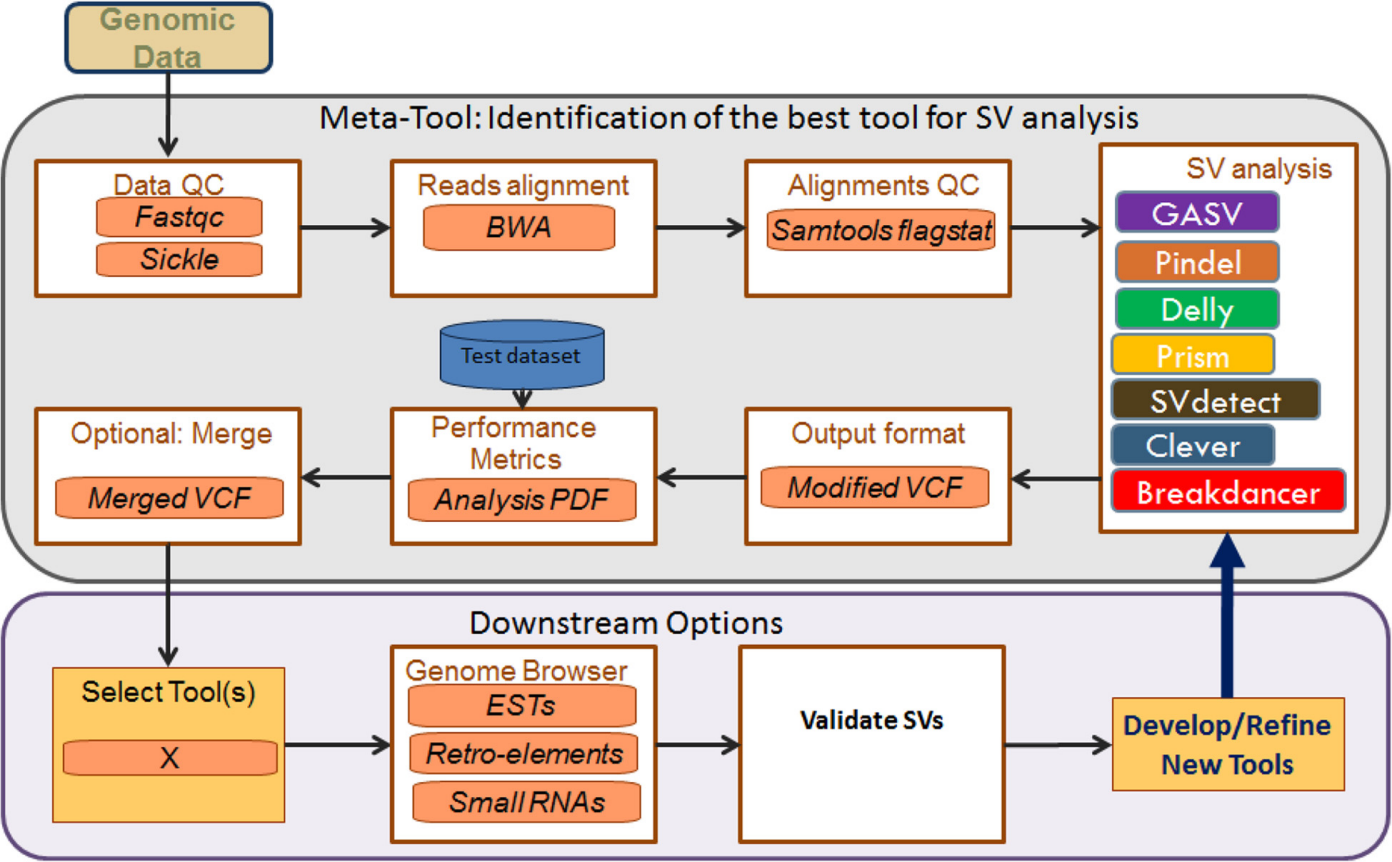
**SV-AUTOPILOT pipeline.** Illumina pair-end NGS data in the form of a fastq file is submitted to the pipeline for SV analysis along with a genomic reference sequence. A quality report is provided by Fastqc, and Sickle is used for trimming low quality reads. Modularity allows for a choice of read aligner and SV tools. Samtools flagstat is run to evaluate the quality of the mapping. Each tool’s output is converted to a VCF format, unless already provided by the program, for downstream use by the researcher. For those wanting to benchmark tool performance, the performance metrics for the tools can be compared in the PDF report provided. Finally, when using multiple tools as part of a pipeline leading to SV validation, the option to merge SV calls according to the statistical method provided here is available to enrich the call set with true calls by merging results and reducing false-positive calls.

While generation of a BAM file from raw reads proceeds sequentially, SV discovery tools may be run in parallel (Figure 1). Additional scripts written in Python have been included to transform the output of the tools into VCF, if needed. Additional parameters can be set for the Performance Metrics analysis and the optional merge step, discussed below. The Makefiles for SV-AUTOPILOT are supported and are available via the github repository (https://github.com/ALLBio/allbiotc2). The pipeline for SV-AUTOPILOT, including pre-processing via FASTQC [39] and Sickle [40], is detailed in Figure 1.

### Merging: statistical considerations

The difficulty in creating a “consensus” call set from different, individual call sets consists in identifying virtually identical calls and merging such calls into one, unifying call. While a few ad-hoc procedures have been suggested in the literature [41,42], neither of them addresses how to control the false discovery rate, that is the amount of calls that are merged mistakenly because of random effects. Moreover, they also do not address the specific strengths and weaknesses of the tools whose usage led to generation of the individual call sets. A statistically sound merging procedure should be guided by two insights:

1. The accuracy of SV breakpoints provided can vary substantially among SV discovery tools. While split-read aligners tend to deliver highly accurate breakpoints, internal segment size based approaches deliver inaccurate breakpoints. This has two implications: first, merging criteria for internal segment size based approaches should be more relaxed and, second, the consensus call should indicate the most accurate breakpoint predictions available.
2. Calls may be mistakenly merged, simply due to random effects, such as fluctuations of call density, too large individual call sets, and so on, and one would like to control the *false discovery rate,* that is, the amount of mistakenly merged events. In other words, merging criteria should be such that randomly chosen call pairs meet them only with low probability.

While 1) can be addressed by evaluating tools on benchmark datasets, 2) needs further elaboration. Consider, for example, two tools that, on a genome of length G, within a certain size range---for example, deletions of size 20–50 bp---have generated call sets of size *K*_1_ and *K*_2_, respectively. That is, *K*_1_ out of G bases in the genome are affected by a breakpoint (for deletions here and in the following: the centerpoint between the left and right breakpoint) of a deletion of size 20–50 bp predicted by the first tool, respectively K_2_ out of G bases are affected by breakpoints (deletions: centerpoints) predicted by the second tool. Let the merging criterion be that the breakpoints of two calls do not deviate by more than L basepairs (see “Note on reciprocal overlap” below, why reciprocal overlap is not a statistically sound criterion, hence should be avoided when merging, and also evaluating calls). The probability ${P}_{{\mathrm{K}}_1,{\mathrm{K}}_{2,}G}$ that the breakpoints of two randomly picked calls, one from the first tool and one from the second tool, are at a distance of at most L basepairs is

$$ {P}_{{\mathrm{K}}_1,{\mathrm{K}}_{2,}G}=1-\left(1-{\left(1-{\left(1-\frac{\mathrm{L}}{\mathrm{G}}\right)}^{K_1}\right)}^{K_2}\right)=1-{\left(1-\frac{\mathrm{L}}{\mathrm{G}}\right)}^{{\mathrm{K}}_1{\mathrm{K}}_2}\approx 1- \exp \left(-{\mathrm{K}}_1{\mathrm{K}}_2\frac{\mathrm{L}}{\mathrm{G}}\right) $$

### Merging: note on reciprocal overlap as criterion

Reciprocal overlap does not represent a sound criterion for merging two calls, and also for evaluating calls, because:

1. For large deletions, breakpoints are allowed to deviate by massive amounts of base pairs. For example, requiring 50% reciprocal overlap for two deletions of 10,000 bp in length allows a distance of 5000 bp between breakpoints. A random caller that randomly places breakpoints in the genome is considerably more likely to place a “good” breakpoint than, for example, when considering 100 bp deletions.
2. For truly small deletions, say of 20 bp in length, breakpoints are only allowed to deviate by at most 10 bp (for the case of 50% reciprocal overlap). This, however, is oblivious to the repetitiveness of many genomes and to the fact that gap placement is difficult, which renders it possible that two different calls are virtually identical although deviating by up to 50 bp in terms of breakpoints.
3. There is no obvious, overlap-based criterion for insertions.

In summary, the idea of using (whatever form of) overlap for merging and evaluating calls is statistically unsound and introduces severe, misleading biases when merging calls.

### Merging: parameters

Guided by the considerations outlined above, we determine that two calls are to be merged if their breakpoints do not deviate by more than 50 bp and the lengths of the indels predicted do not deviate by more than 20 bp. While the merging algorithm is able to take tool-specific criteria into account, we found that the unifying criteria in use here yielded excellent results in our benchmark. As a general guideline for adapting criteria to tools, we recommend stricter criteria for split-read aligners (for example, 20 bp distance and 10 bp length deviation), because (split-)alignment based breakpoint predictions tend to be very accurate (while still being prone to misplacement due to repetitive sequence and gap placement artifacts) whereas for IS based approaches 100 bp distance and 100 bp length deviation are still the most sensible, because on top of the usual issues due to repetitive sequence, these tools can predict highly inaccurate indel breakpoints.

It is worth noting that the probability that the breakpoints of two calls do not deviate by more than 50 bp is never larger than 0.01, for any of the tools and genomes considered here when merging calls whose lengths do not deviate by more than 20 bp---which determines the sizes and K_1_ and K_2_ of two different call sets to be compared, as computed by the above formula. Therefore, criteria of this order of magnitude (i.e., allowing differences of tens of basepairs) are a good general choice when studying within-species genetic variation.

### Merging: algorithm

The merging algorithm receives calls (insertions or deletions) from different tools, all of which are specified by a breakpoint and the length of the variant in question (for insertions: the base position where the new sequence has been inserted; for deletions: the center point between the left and the right base position that specify the boundaries of the deleted sequence. *Note:* specifying the center-point and the length of a deletion uniquely determines a deletion. As discussed above, the merging algorithm merges calls that are significantly close to each other, such that both calls are statistically likely (significantly) to have discovered the same insertion or deletion in the genome under consideration.

As a formal model to capture this, consider a graph, in the sense of graph theory. The nodes of the graphs are the calls from the different tools, and an edge between two calls reflects that the breakpoints are at a distance of not more than 50 bp and that the length does not deviate by more than 20 bp, which, as per the considerations from above, translates into statistical evidence that the two calls correspond to the same indel.

After having constructed this ‘call graph’, all of its maximal cliques are identified. We recall that, by definition, a clique is a subset of nodes all of which are pairwise connected by edges. Hence, a clique translates into a set of calls all of which are statistically likely to represent virtually identical variants. Maximal cliques, that is cliques to which no further nodes can be added without violating the clique property, represent maximal sets of calls that point at the same, likely correct indel. Hence, they are maximal call subsets that one should merge into one unifying call.

Enumerating all maximal cliques proceeds by making use of an algorithm that greatly profits from the fact that calls can be ordered by their breakpoints in a left-to-right fashion. The algorithm was successfully used in other graph-based settings where nodes specified genomic loci and could be ordered in a left-to-right fashion [15]. The merging algorithm is implemented in Python. The algorithm is very fast; for example, using a MacBookPro5,5 (2.53 GHz Intel Core 2 Duo processor), it merges call sets from as many as 8 tools within only 2 or 3 minutes.

### Benchmarking: comparative analysis report

In the final step of the SV-AUTOPILOT pipeline, the predictions of each tool are compared to the true annotations if available. In this, the considerations are similar to those used for merging. That is, we take into account that the breakpoint and length specifications of tools can deviate from the true annotations, even though they have indeed discovered the true indel in question. Reasons for this are plentiful. As expected, internal segment size based approaches are unable to specify breakpoints (highly) accurately. Even (split) alignment based approaches may fail to provide accurate breakpoints, as accurate gap placement in alignments has remained an algorithmic challenge in bioinformatics (see e.g. [26] for a description of effects such as gap annihilation, gap wander and so on).

A predicted insertion/deletion is considered as a match to a true insertion/deletion if the distance of their center points and their length difference are below user defined thresholds. When choosing thresholds, the tools’ characteristics, the genome under investigation, and the insert size distribution of the sequencing library should be taken into consideration. As discussed above, split-read methods tend to be more accurate than paired-end methods in terms of breakpoint resolution. A large standard deviation of the sequencing library will lead to read pair methods being less accurate when estimating the length of an indel.

Here, two different sets of parameters were used, which we refer to as *strict* and *relaxed.* The strict parameters require a center distance of at most 50 bp and a length difference of at most 20 bp, while the relaxed criteria ask for a center distance of at most 100 bp and a length difference of at most 100 bp. Both definitions still ensure that a match is statistically significant (i.e. unlikely to occur just by chance), which has been guided by considerations that are similar to the ones we have described for merging call pairs -- the difference here is that one of the call sets are the true annotations. The relaxed setting was included to show that some tools make calls that are near true events but don’t exactly hit them (as indicated by the difference between strict and relaxed precision). Based on these criteria, the analysis scripts reports various different statistics stratified by length range. The above parameters were used in benchmarking; however, these can be modified by SV-AUTOPILOT users and adapted to their dataset and tool set.

To assess general performance, the absolute number of calls as well as precision and recall are reported. *Precision* is the percentage of predictions that match a true annotation and thus measures the fidelity of the calls made by a given tool. *Recall*, on the other hand, is the percentage of true events that have been spotted by the given tool and thus measures the comprehensiveness of the delivered call set. Reporting these two performance statistics allows researchers to choose a tool that suits their needs. For instance, when seeking to discover new variants, high recall may be more important than high precision so as to capture as many true calls as possible. Conversely, when validation is planned, a low false positive rate is imperative and the focus of SV detection would be on high precision. Following validation, realignment may be performed and the preference may again change to that of a high recall rate for the purposes of SV discovery, knowing you may encounter a higher number of false positives. For the purposes of presenting an overall tool performance metric, the F-measure is provided, defined as 2*precision*recall/(precision + recall). Thus, this measure integrates recall and precision into one single performance indicator.

Some false positive predictions of insertions or deletions are caused by *substitution (or mixed) events* where a stretch of DNA in the reference genome has been replaced by another piece of DNA in the donor genome under study. The lengths of the deleted and the inserted parts need not be equal and we refer to their difference as the *effective length* of a substitution event. Internal segment size based approaches are especially prone to confusing such events with insertions or deletions of the same effective length. Therefore, the reports provided by SV-AUTOPILOT contain another column (*Mix.*) with the percentage of predictions that do not match an insertion/deletion but do match a true mixed event of similar effective length.

To assess the accuracy of each tool in terms of the reported breakpoint positions, we also report the average center point distance as well as the average length difference of all predictions that match a true event. Knowing the accuracy in terms of breakpoint coordinates can be valuable for correctly merging calls of different tools and for the design of primers used in SV validation.

### Interpretation of the results with Radarplots

**Figure 2:**
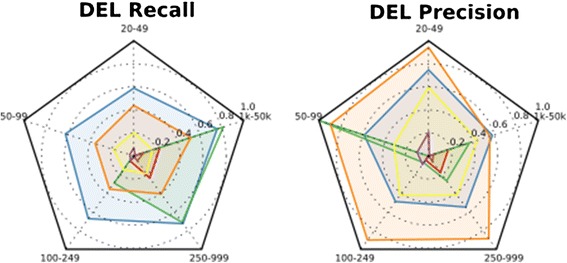
**Radar plot interpretation.** Each corner of the pentagon represents a size class of SV. Performance is measured on a scale of 0–1.0, with 1.0 as the most accurate calls. Each tool is associated with a color as indicated in the associated figure legend. Tool performance across all size classes is easily assessed by evaluating the total area of the radar plot covered by a given tool.

To ease the interpretation of the performance metrics, radarplots, generated using matplotlib [43], are plotted providing the measurements of tool performance for *Recall, Precision,* and the *F-measure*. Some tool types are expected to perform better than others for a given metric. In general, it is expected that split read aligners will be more accurate at reporting breakpoints and thus perform with higher precision than other types of aligners. The radar plots provided here are in the shape of a pentagon (Figure 2). Each angle of the pentagon relates to a size class of SV evaluated. Tool performance data is plotted for each size class and the points are connected to provide a visual representation of performance across all size classes.

## Results and discussion

The SV-AUTOPILOT pipeline was created to provide a “meta-tool” platform for using multiple SV-tools, to standardize benchmarking of tools, and to provide an easy, out-of-the-box SV detection program. As most SV tools have been designed, and/or optimized for performance on human datasets, benchmarking tool performance on another organism was needed to determine whether some tools are more adaptable to a non-human genome than others. As the field of SV detection continues to develop and evolve, and more tools continue to become available, a standardized method of evaluating their performance relative to other existing tools was also needed. Here we show that SV-AUTOPILOT addresses each of these needs.

SV-AUTOPILOT was used to benchmark seven Structural Variation (SV) prediction tools. The tools were tested on their ability to identify SVs in the two reconstructed genomes described, human chromosome 21 and the Arabidopsis Lansberg (Ler) genome, a plant genome. Human chromosome 21 was chosen as a representative sample of the human genome which is small in size and for which many structural variants have been validated. As most SV tools were designed for use with the human genome, it is expected that tools will perform well on the human dataset and that it can be used in a comparison of tool performance on the Arabidopsis genome. For reports on tool performance on the human genome as a whole, please see the original SV tool publications. In addition, we have provided the results of the entire Venter genome data as an example of a larger, whole genome run in the Additional file 1. As expected, the tools perform much the same, however some tools were unable to meet the demands of managing such a large genome, likely due to the higher memory needs for SV processing.

The tools included in the SV-AUTOPILOT virtual machine and which were used for benchmarking included GASV, Delly, Breakdancer, Pindel, Clever, SVDetect, and Prism. Due to the modularized set-up employed by SV-AUTOPILOT, SV tools can be easily added or removed from the pipeline. In addition, the user is able to choose which of several alignment algorithms is used in their analysis. For example, in the initial testing phase, both BWA-mem and Bowtie2 were tested for each tool. BWA-mem was chosen for continued downstream use in the benchmarking as all the tools tested performed a few percentage points higher in recall and precision (data not shown). For the purposes of benchmarking, the ability of a tool to accurately call an SV was measured using the analysis tools described and reports were generated for both Ler and Human Chromosome 21 data at standard deviations of 15 and 50 (provided in Additional file 1).

### Performance metrics

Often researchers are unable to predict the computing requirements for a given tool as this information may be missing in the literature or beyond the reach of the average biologist. For those required to make specific requests for computing time at computing centers or on the Cloud, this can be a severe bottleneck to initiating their research. To facilitate future analysis of tool performance, each tool in this study was evaluated for its computing performance (Table 3) in addition to SV prediction assessment. Data was collected for RAM and CPU usage, run time, as well as IO and threading abilities.

**Table 3:** **Typical computational performance by SV tools used for a single run**

**Tool**	**Multi- threading**	**Mem use on Tair (Mb)**	**Mem use on Human (Mb)**	**CPU time Tair (h:m.s)**	**CPU time Human (h:m.s)**	**Algorithm**	**SV’s**
GASV	n	1058	594	0:02.08	0:01.20	PE	IDVT
Delly	n	578	1236	0:15.02	0:03.18	PE & SR	DVTP
Breakdancer	n	21.9	7	0:02.41	0:27.7	PE	IDVT
Pindel	y	3500.5	5779	3:02.46	1:16.0	SR	IDVP
Clever	y	238.7	1598	0:15.47	0:14.04	PE	ID
SVdetect	n	172.3	3223	0:07.56	0:07.31	PE	IDVTP
Prism	n	1024.9	6817	0:28.15	0:05.59	PE & SR	IDVP

Log files document computation performance for each tool used in this benchmarking study. Documentation from a single run shows memory (mem) usage and CPU time need to run each tool on Arabidopsis (Tair) and on the Human dataset used in the benchmarking. Additional columns refer to the type of algorithm used (PE: Paired-end; SR: Split-read) and the SVs that the tool is reported to be able to predict (I: Insertion; D: Deletion; V: Inversion; T: Translocation; P: Duplication). Raw log files are included in the supplementary data.

Each tool was run independently on the Arabidopsis dataset and evaluated for computational performance. As shown in Table 3, CPU time varied widely across tools. For example, GASV completed the run in 2 minutes, while Pindel took 3 hours and 3 minutes to run. Clever and Pindel both allow for parallelization while the others tools run on a single thread. Although parallelization generally improves performance, Pindel does not appear to show the gains expected. This may be due to the large volume of calls made by Pindel in addition to Pindel’s very verbose VCF file.

### Prediction performance

The ability of each SV tool to accurately call an SV event was evaluated and a PDF report produced as part of SV-AUTOPILOT’s pipeline (see Additional file 1). While there are multiple ways of defining ‘accuracy’, in statistics it is typically defined as (true positive + true negative)/(true positive + true negative + false positive + false negative). As ‘true negatives’ are not applicable in this setting, we choose to use an integrative measure of recall and precision, provided here as the F-measure. In a biological context, ‘accuracy’ can refer to a measure of how close the breakpoint predictions of true positive calls were to the true breakpoint coordinates. Therefore we provide the average length difference and the center point distance between true call and prediction. We have separated statistics into ‘strict’ and ‘relaxed’ categories which implicitly address questions of tool accuracy.

Each length class of SV was examined individually to evaluate performance. Table 4 provides an overview of performance by each of the tools on ideal (sd = 15) and less ideal (sd = 50) datasets for Arabadopsis and Human Chromosome 21. When examined for Precision, the ability to match a true insertion/deletion, and Recall, percentage of predicted true insertion/deletions out of total true SVs provided in the dataset, a few tools were shown to be more adaptable to the Arabidopsis dataset than the others: Clever and Pindel, with Delly performing well in the largest size class (>1000 bp).

**Table 4:** **Best performing SV tool for each size class of insertion and deletion using normal and less-ideal datasets of Arabidopsis and Human Chromsome 21**

***A. Precision***							
	**Data type**	**Std Dev**	**Length 20–49 P (rel/str)**	**Length 50–99 P (rel/str)**	**Length 100–249 P (rel/str)**	**Length 250–999 P (rel/str)**	**Length 1 K-50 K P (rel/str)**
	***Insertion***						
	Human chr. 21	15	Clever	Pindel	Clever	Clever	n/a
			88.3/77.8	85.7/68.8	88.5/45.8	100.0/50	
	Tair v.9	15	Pindel	Clever/ Pindel	Clever	Clever	n/a
			95.0/93.2	94.3/47.4	68.1/23	66.7/55.6	
	Human chr. 21	50	Pindel	Clever/Pindel	Clever	n/a	n/a
			87.9/75.2	83.3/55.6	66.7/17.9		
	Tair v.9	50	Pindel	Clever/Pindel	Clever	Clever	n/a
			94.9/92.7	94.6/46.2	73.9/8.3	60.0/20	
	***Deletion***						
	Human chr. 21	15	Clever/Pindel	Pindel	Clever/Pindel	Pindel	Breakdancer/Clever
			92.8/89	92.3/76.9	84.6/42.9	100/100	100/33.3
	Tair v.9	15	Pindel	Delly	Pindel	Pindel	Clever
			94.6/94.2	100/100	89.2/90.2	87.9/88.7	68.3/58.1
	Human chr. 21	50	Clever/Pindel	Pindel	Clever/Pindel	Pindel	Breakdancer/Clever
			90.9/88.2	100/90	59.1/42.9	81.8/81.8	100/50
	Tair v.9	50	Pindel	Delly/Pindel	Pindel	Pindel	Clever/Pindel
			94.5/94.3	100/90.6	86.5/86.3	89.4/89.4	69.6/61
***B. Recall***							
	**Data type**	**Std Dev**	**Length 20–49 R (rel/str)**	**Length 50–99 R (rel/str)**	**Length 100–249 R (rel/str)**	**Length 250–999 R (rel/str)**	**Length 1 K-50 K R (rel/str)**
	***Insertion***						
	Human chr. 21	15	Clever	Clever	Clever	Clever	n/a
			72.8	83.3	60	5.3	n/a
	Tair v.9	15	Clever	Clever	Clever	Clever	n/a
			56.4	81.2	92.7	15.9	n/a
	Human chr. 21	50	Pindel	Clever	Clever	Clever	n/a
			61.8	48.6	76.7	10.5	n/a
	Tair v.9	50	Pindel	Pindel	Clever	Breakdancer	n/a
			34.6	43	95.1	51.2	n/a
	***Deletion***						
	Human chr. 21	15	Prism	Clever	Clever	Delly	Breakdancer
			88.1	90.9	52.6	73.7	50
	Tair v.9	15	Clever	Clever	Clever	Delly	SVDetect
			65	82.1	89.8	93	97
	Human chr. 21	50	Prism	Prism	Clever	Breakdancer	Breakdancer
			85.6	72.7	63.2	73.7	50
	Tair v.9	50	Pindel	Clever	Clever	Delly	SVDetect
			94.5	52.9	91.9	94.9	96.2

For this work, a standard deviation of 15 is considered normal while a standard deviation of 50 is considered less-ideal. Recall and Precision were two measures used to evaluate the ability of a tool to accurately predict SVs. Here the winner for each length class is provided along with the tools winning value for that category. (P = Precision; R = Recall; Std. Dev = Standard Deviation of the Insert size; n/a = no call was made by any tools tested). The Additional file 1 contains all tool performance statistics in the PDF reports. In Table 4a both ‘relaxed’ and ‘strict’ criteria (REL/STR) (see Methods) are provided for the precision measurements which indicates how accurate the tools are at making their calls. In Table 4b the scores of tool recall demonstrate how much of the SVs the tools are able to discover**.**

### Effect of different standard deviations

In the purification of ligation product step of llumina paired-end sample preparation protocol, different settings may cause the resulting DNA fragment library to have an insert library with variations in standard deviation (sd) of the insert size [12]. Therefore simulated reads for each sample were prepared at both sd 15 and 50.

**Figure 3:**
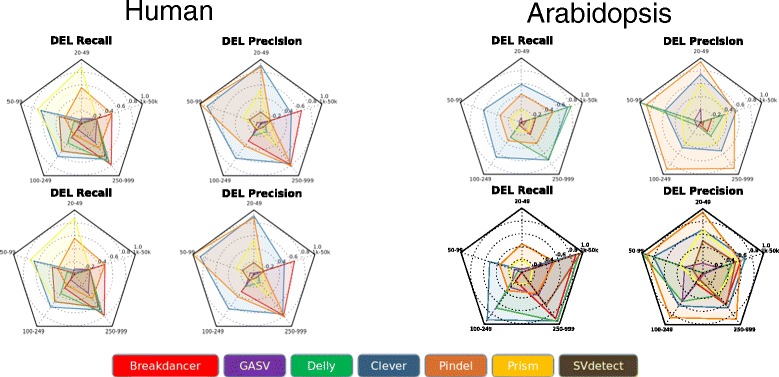
**Data quality affects the performance of SV tools in human and Arabidopsis data sets**. Some tools are more affected by changes in data quality than others. The standard deviation of the insert size of paired end reads was used as a measure of data quality. The Recall and Precision of Deletion calls are measured for Human and Arabidopsis datasets at the less optimal (sd = 50) and more optimal standard deviation (sd = 15).

In the low quality (sd = 50) Arabidopsis dataset, Pindel outperformed other tools in precision and recall at SV lengths ranging from 20–49, whereas in human chromosome 21, Clever showed better precision and recall. At the better standard deviation of 15, Clever consistently performed with the best recall in all SV length classes (Figure 3).

Unlike the other tools, Delly showed no change in calling ability between the sd 15 and sd 50 datasets. Delly consistently excelled at recall in the largest SV classes of deletions (>1000 bp). For other tools the change in sd resulted in more significant changes in performance. For instance, Breakdancer’s performance dropped considerably in the lower quality dataset, especially for the smaller size classes. However, this is consistent with Breakdancer documentation [11].

### Adapting to non-human datasets, benchmarking in Arabidopsis

Tools tested in this study were initially developed for use in human genome analysis. However, with the reduction in sequencing costs, many non-human genomes are being sequenced to identify SVs. Arabidopsis is a well-studied plant species with many validated SVs. In this analysis we compared the ability of various tools to accurately call SVs in the genome of a species for which it was not initially designed.

Only 4 of the tools tested were able to predict insertions: BreakDancer, Pindel, Clever and SVDetect. However, although SVDetect made a few predictions of insertions in the human dataset, it did not make any predictions at all in the Arabidopsis dataset regardless of sequence library insert size standard deviation. This was unusual as although more tools performed at sd 15 than 50, all the other tools were able to make predictions for both genomes.

As most tools performed better overall on the datasets with a smaller standard deviation, the comparison of tool performance, between Human Chromosome 21 and Arabidopsis, provided here is limited to the sd 15 datasets. When compared to the human chromosome 21 dataset, SV tools performance on the Arabidopsis dataset showed reduced precision of calls in size classes spanning from 50-99 bp and 100-249 bp, but higher recall over all size classes among insertion events. When examining deletions, the Arabidopsis dataset had comparable recall to the human dataset at 50-99 bp, but was reduced in the class 20-49 bp. Above 100 bp, the Arabidopsis data set performed with higher recall than the human dataset, however, it is possible that the limited number of validated events in the human chromosome 21 dataset in this size class may limit the statistical power of this analysis. When examining calls on the basis of recall and precision, Pindel and Clever consistently out-performed other callers. In general, Clever had higher overall recall while Pindel was found to perform with higher precision in its calls. However, Prism often performed with better recall in the human dataset, but not in Arabidopsis. Interestingly, Delly showed the highest recall in the largest size class of Arabidopsis. This suggests that Pindel and Clever may be able to offer the best calls for non-human datasets with Delly being useful for identifying deletions in the largest size class.

### Meta-tool performance

Benchmarking of the Arabidopsis dataset used here has shown that some SV tools may be more adaptable in working with a non-human dataset than others. As demonstrated here, tools may vary considerably in their performance depending on the size class of SVs to be identified as well as the quality of the genomic reads. Therefore, it is clear that a meta-tool is needed to not only provide SV calls, but to group the call-sets from multiple SV tools and provide a filtered output based on the performance metrics of the individual tools. In SV-AUTOPILOT, a merging script was developed to take all the calls, filter them, and provide a merged output (provided in Additional file 1). This merging may be initiated by the user following the completion of all SV tool runs and parameters set to tailor the output according to user preferences pertaining to recall and precision.

Unlike other SV merging tools, here researchers receive fewer and more accurate calls to begin their investigations to validate SVs. When Arabidopsis (sd = 15) calls were filtered through the merge script, total calls were reduced by as much as 70% (average 61%) with recall diminished, on average, 27%. In merging a small portion of recall is lost for the benefit of a reduction of more than half of the calls total (due to merging and elimination of many false-positives). This results in an enriched set of true calls in the call set for validation.

**Figure 4:**
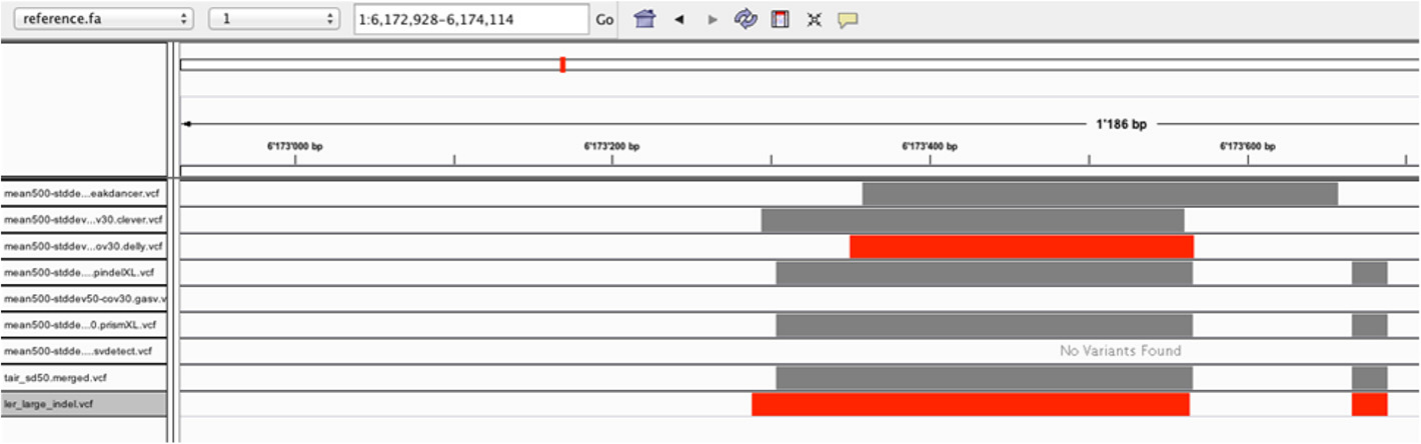
**Example of merged call set compared to individual call sets.** Integrated Genome Browser view of merged predictions. Calls made by each tool are shown in individual tracks, and the merged call set provided by SV-AUTOPILOT is shown in the bottom track. In this example a larger call by Breakdancer has been recentered by the merging algorithm. The red lines on the bottom indicate the position of the reference variants.

As shown in Figure 4, the merging script is able to cluster calls by evaluating their breakpoints relative to the tool algorithm type applied (see methods section for a complete description). The user may select which VCF outputs from the various tools run in the SV-AUTOPILOT pipeline to be considered in the merging process. Merged call-sets, as with the other tools included in the pipeline, are provided in VCF format and can be visualized on any genomic browser (however, indexing and/or a track definition file may be required).

### Tool use and development

The SV-AUTOPILOT meta-tool is packaged as a VM. This allows for standardization of the pipeline for SV prediction. SV tool benchmarking on new genomes, and provides a platform for evaluating the performance of SV tools under development. The SV-AUTOPILOT virtual machine is packaged with 7 of the most popular SV tools currently available, reflecting the most recent updates and versions (as of January 2014). In addition, a merge script has been added which will filter and rank calls providing researchers with an accurate and condensed set of calls curated from all the tools. For the purposes of this work, SV-AUTOPILOT has been run in a whole with all SV-modules enabled. Default configuration of the pipeline is set to the pipeline described for the benchmarking in this work, however, it is also customizable to allow for a tailored analysis.

Each of the modules, both SV-callers and pre- and post-processing steps, can be modified by changing the predefined running parameters while maintaining the structure. A general pipeline config file hosts the configuration for the pipeline. Project specific or sample specific settings, involving file conventions, locations of genome files and program definitions can be altered from the invocation. More specific settings can be changed or a new procedure added into the pipeline (Detailed instructions can be found in Additional file 1).

For example, the alignment algorithm used in this project was BWA-mem [23]. The modularized design of SV-AUTOPILOT allows for other aligners, currently provided in the modules section (bowtie1, bowtie2, bwa-backtrack, bwa-mem, stampy), to be used. This allows a comparative study of different settings or versions of software using the same reproducible setup. Indeed, we cannot claim that our decision to do alignment with BWA-mem is the best practice now or in the future. Therefore the pipeline allows replacement of functional components with alternative implementations. It is our intention that this tool be used not only by researchers for generating comprehensive SV calls, but also by programmers/developers for the testing performance of tools they are developing against currently available tools. SV-AUTOPILOT is packaged in a VM and allows for just that. The tool is available at https://bioimg.org/sv-autopilot.

## Conclusions

Here we have taken ‘human’ SV prediction technology and applied it to a non-human organism, Arabidopsis. We have provided benchmarking data on the performance of seven of the most popular SV prediction tools and tested them on reads of varying quality. Tools were shown to vary in their ability to adapt to a non-human genomic dataset and datasets of varying quality.

This work demonstrates the importance of using multiple SV tools in order to cover a wide range of SV size classes and to minimize false-positive calls. We have packaged several of these tools into a single Virtual Machine pipeline, SV-AUTOPILOT, to facilitate reproducible research and coupled that with a powerful merging script that filters and ranks calls providing researchers with an accurate dataset on which to begin their bench validations. All call-sets have been formatted to fit the standard VCF format to facilitate visualization in genomic browsers and interoperability with other genomic tools. SV tool developers and SV researchers are able to test new tools against existing tools to examine performance, evaluate new datasets for tool optimization, and to generate a high quality enriched set of SV calls for further validation. The SV-AUTOPILOT VM can be downloaded with all datasets, tools and algorithms presented here at https://bioimg.org/sv-autopilot.

## Additional file

Additional file 1:
**The data sets supporting the results of this article are available in the as part of the SV-AUTOPILOT virtual machine, in** https://bioimg.org/sv-autopilot **.** The scripts used as the basis for the virtual machine described in this article are available via the GitHub repository, in https://github.com/ALLBio/allbiotc2/.
