# Supplementary material for: SV-AUTOPILOT: optimized, automated construction of structural variation discovery and benchmarking pipelines
Source: BMC Genomics. 2015 Mar 25;16(1):238. doi: 10.1186/s12864-015-1376-9 (PMC4520269; doi:10.1186/s12864-015-1376-9)
Supplement: Additional file 1: — The data sets supporting the results of this article are available in the as part of the SV-AUTOPILOT virtual machine, in https://bioimg.org/sv-autopilot . The scripts used as the basis for the virtual machine described in this article are available via the GitHub repository, in https://github.com/ALLBio/allbiotc2/. [file 12864_2015_1376_MOESM1_ESM.zip › 1993348534130930_add1.pdf]

1 Command line

```
../../allbiotc2/evaluation/evaluate-sv-predictions2 -R 20-49,50-99,100-249,250-999,1000-50000 -e
    human_sd15_o50z20 -o 50 -z 20 -L ../../data/reference_human/venter.phased.b37.chr21.nodots.vcf
    mean500-stddev15-cov30.breakdancer.vcf mean500-stddev15-cov30.clever.vcf mean500-stddev15-cov30.
    delly.vcf mean500-stddev15-cov30.gasv.vcf mean500-stddev15-cov30.pindel.vcf mean500-stddev15-cov30.
    prism.vcf mean500-stddev15-cov30.svdetect.vcf
```

2 Overall performance

2.1 Insertions

	Abs.	Prec.	Mix.	Rec.	Exc.	F.	ΔLen.	Dist.
**Length Range 20–49** (136 true insertions)								
m500-sd15-cov30.breakdancer	0	–	–	0.7	0.7	–	–	–
m500-sd15-cov30.clever	137	**77.4**	0.7	**58.8**	**20.6**	**66.8**	5.5	17.0
m500-sd15-cov30.delly	0	–	–	0.0	0.0	–	–	–
m500-sd15-cov30.gasv	0	–	–	0.0	0.0	–	–	–
m500-sd15-cov30.pindel	117	76.1	**0.9**	56.6	18.4	64.9	**1.5**	**4.7**
m500-sd15-cov30.prism	0	–	–	0.0	0.0	–	–	–
m500-sd15-cov30.svdetect	1	0.0	0.0	0.0	0.0	–	–	–
**Length Range 50–99** (37 true insertions)								
m500-sd15-cov30.breakdancer	62	1.6	0.0	0.0	0.0	0.0	17.0	**1.0**
m500-sd15-cov30.clever	35	45.7	**2.9**	**40.5**	**21.6**	**43.0**	3.6	17.6
m500-sd15-cov30.delly	0	–	–	0.0	0.0	–	–	–
m500-sd15-cov30.gasv	0	–	–	0.0	0.0	–	–	–
m500-sd15-cov30.pindel	14	**71.4**	0.0	18.9	2.7	29.9	**0.1**	8.1
m500-sd15-cov30.prism	0	–	–	0.0	0.0	–	–	–
m500-sd15-cov30.svdetect	4	25.0	0.0	2.7	0.0	4.9	11.0	18.0
**Length Range 100–249** (30 true insertions)								
m500-sd15-cov30.breakdancer	26	0.0	**0.0**	0.0	0.0	–	–	–
m500-sd15-cov30.clever	26	**38.5**	**0.0**	**33.3**	**33.3**	**35.7**	**4.6**	**21.8**
m500-sd15-cov30.delly	0	–	–	0.0	0.0	–	–	–
m500-sd15-cov30.gasv	0	–	–	0.0	0.0	–	–	–
m500-sd15-cov30.pindel	0	–	–	0.0	0.0	–	–	–
m500-sd15-cov30.prism	0	–	–	0.0	0.0	–	–	–
m500-sd15-cov30.svdetect	3	0.0	**0.0**	0.0	0.0	–	–	–
**Length Range 250–999** (19 true insertions)								
m500-sd15-cov30.breakdancer	5	0.0	**0.0**	0.0	0.0	–	–	–
m500-sd15-cov30.clever	1	**100.0**	**0.0**	**5.3**	**5.3**	**10.0**	**6.0**	**3.0**
m500-sd15-cov30.delly	0	–	–	0.0	0.0	–	–	–
m500-sd15-cov30.gasv	0	–	–	0.0	0.0	–	–	–
m500-sd15-cov30.pindel	0	–	–	0.0	0.0	–	–	–
m500-sd15-cov30.prism	0	–	–	0.0	0.0	–	–	–
m500-sd15-cov30.svdetect	0	–	–	0.0	0.0	–	–	–
**Length Range 1000–50000** (10 true insertions)								
m500-sd15-cov30.breakdancer	0	–	–	**0.0**	**0.0**	–	–	–
m500-sd15-cov30.clever	0	–	–	**0.0**	**0.0**	–	–	–
m500-sd15-cov30.delly	0	–	–	**0.0**	**0.0**	–	–	–
m500-sd15-cov30.gasv	0	–	–	**0.0**	**0.0**	–	–	–
m500-sd15-cov30.pindel	0	–	–	**0.0**	**0.0**	–	–	–
m500-sd15-cov30.prism	0	–	–	**0.0**	**0.0**	–	–	–
m500-sd15-cov30.svdetect	0	–	–	**0.0**	**0.0**	–	–	–

2.2 Deletions

	Abs.	Prec.	Mix.	Rec.	Exc.	F.	ΔLen.	Dist.
**Length Range 20–49** (118 true deletions)								
m500-sd15-cov30.breakdancer	0	–	–	0.8	0.0	–	–	–
m500-sd15-cov30.clever	111	82.9	**0.9**	74.6	5.9	**78.5**	7.3	17.0
m500-sd15-cov30.delly	0	–	–	0.0	0.0	–	–	–
m500-sd15-cov30.gasv	64	29.7	0.0	10.2	0.0	15.1	9.5	37.5
m500-sd15-cov30.pindel	82	**90.2**	0.0	61.9	0.0	73.4	**0.3**	**1.6**
m500-sd15-cov30.prism	270	42.6	0.4	**86.4**	**6.8**	57.1	1.1	3.5
m500-sd15-cov30.svdetect	12	8.3	0.0	0.8	0.0	1.5	14.0	13.0
**Length Range 50–99** (33 true deletions)								
m500-sd15-cov30.breakdancer	85	1.2	0.0	0.0	0.0	0.0	20.0	47.0
m500-sd15-cov30.clever	29	65.5	**3.4**	**63.6**	9.1	**64.6**	7.6	20.5
m500-sd15-cov30.delly	0	–	–	3.0	0.0	–	–	–
m500-sd15-cov30.gasv	11	45.5	0.0	18.2	3.0	26.0	4.8	34.2
m500-sd15-cov30.pindel	13	**76.9**	0.0	27.3	0.0	40.3	**0.4**	4.9
m500-sd15-cov30.prism	105	23.8	0.0	60.6	**12.1**	34.2	2.7	**4.8**
m500-sd15-cov30.svdetect	10	40.0	0.0	12.1	0.0	18.6	10.8	12.6
**Length Range 100–249** (19 true deletions)								
m500-sd15-cov30.breakdancer	14	28.6	0.0	**21.1**	**15.8**	24.2	3.0	27.8

m500-sd15-cov30.clever	13	38.5	0.0	**21.1**	10.5	**27.2**	8.0	12.4
m500-sd15-cov30.delly	79	3.8	1.3	10.5	5.3	5.6	8.7	8.0
m500-sd15-cov30.gasv	8	0.0	0.0	0.0	0.0	–	–	–
m500-sd15-cov30.pindel	7	**42.9**	0.0	15.8	0.0	23.1	**0.0**	**0.0**
m500-sd15-cov30.prism	16	18.8	**6.2**	15.8	5.3	17.1	**0.0**	**0.0**
m500-sd15-cov30.svdetect	17	5.9	0.0	5.3	0.0	5.6	1.0	12.5
**Length Range 250–999** (19 true deletions)								
m500-sd15-cov30.breakdancer	15	26.7	**0.0**	21.1	0.0	23.5	3.2	34.1
m500-sd15-cov30.clever	16	75.0	**0.0**	**63.2**	0.0	**68.6**	4.1	15.0
m500-sd15-cov30.delly	94	10.6	**0.0**	52.6	0.0	17.7	**0.0**	1.0
m500-sd15-cov30.gasv	2216	0.1	**0.0**	10.5	**10.5**	0.2	4.0	25.5
m500-sd15-cov30.pindel	9	**100.0**	**0.0**	47.4	0.0	64.3	**0.0**	**0.0**
m500-sd15-cov30.prism	36	33.3	**0.0**	**63.2**	0.0	43.6	**0.0**	0.2
m500-sd15-cov30.svdetect	19	5.3	**0.0**	5.3	0.0	5.3	1.0	7.5
**Length Range 1000–50000** (4 true deletions)								
m500-sd15-cov30.breakdancer	2	0.0	**0.0**	0.0	0.0	–	–	–
m500-sd15-cov30.clever	4	**25.0**	**0.0**	25.0	0.0	**25.0**	7.0	0.5
m500-sd15-cov30.delly	12	16.7	**0.0**	**50.0**	**25.0**	**25.0**	3.0	6.5
m500-sd15-cov30.gasv	4	**25.0**	**0.0**	25.0	**25.0**	**25.0**	2.0	20.0
m500-sd15-cov30.pindel	10	10.0	**0.0**	25.0	0.0	14.3	**0.0**	**0.0**
m500-sd15-cov30.prism	5	20.0	**0.0**	25.0	0.0	22.2	**0.0**	**0.0**
m500-sd15-cov30.svdetect	9	0.0	**0.0**	0.0	0.0	–	–	–

2.3 Table Legend

- **Abs.:** *Absolute number* of predictions made in this length range
- **Prec.:** *Precision*, the percentage of predictions in that length range that match a true deletion/insertion.
- **Mix.:** Percentage of predictions that don't match a true insertion/deletion but a *mixed insertion/deletion event* of the same/similar effective length.
- **Rec.:** *Recall*, the percentage of true insertions/deletions in that length range that have been discovered.
- **Exc.:** *Exclusive calls*: percentage of true insertions/deletions that are *only* discovered by this tool.
- **F:** *F-Measure*: $2 \cdot \text{precision} \cdot \text{recall}/(\text{precision} + \text{recall})$. This integrates precision and recall into one statistic.
- **ΔLen.:** *Length difference*: average length difference between prediction and true insertion/deletion (averaged over all predictions that match a true annotation)
- **Dist.:** *Distance*: average center distance between prediction and true insertion/deletion (averaged over all predictions that match a true annotation)
