# Supplementary material for: SV-AUTOPILOT: optimized, automated construction of structural variation discovery and benchmarking pipelines
Source: BMC Genomics. 2015 Mar 25;16(1):238. doi: 10.1186/s12864-015-1376-9 (PMC4520269; doi:10.1186/s12864-015-1376-9)
Supplement: Additional file 1: — The data sets supporting the results of this article are available in the as part of the SV-AUTOPILOT virtual machine, in https://bioimg.org/sv-autopilot . The scripts used as the basis for the virtual machine described in this article are available via the GitHub repository, in https://github.com/ALLBio/allbiotc2/. [file 12864_2015_1376_MOESM1_ESM.zip › 1993348534130930_add10.pdf]

1 Command line

```
../../allbiotc2/evaluation/evaluate-sv-predictions2 -R 20-49,50-99,100-249,250-999,1000-50000 -e
    human_sd50_o100z100 -o 100 -z 100 -L ../../data/reference_human/venter.phased.b37.chr21.nodots.vcf
    mean500-stddev50-cov30.breakdancer.vcf mean500-stddev50-cov30.clever.vcf mean500-stddev50-cov30.
    delly.vcf mean500-stddev50-cov30.gasv.vcf mean500-stddev50-cov30.pindel.vcf mean500-stddev50-cov30.
    prism.vcf mean500-stddev50-cov30.svdetect.vcf
```

2 Overall performance

2.1 Insertions

	Abs.	Prec.	Mix.	Rec.	Exc.	F.	ΔLen.	Dist.
**Length Range 20–49** (136 true insertions)								
m500-sd50-cov30.breakdancer	0	–	–	0.0	0.0	–	–	–
m500-sd50-cov30.clever	0	–	–	5.9	2.2	–	–	–
m500-sd50-cov30.delly	0	–	–	0.0	0.0	–	–	–
m500-sd50-cov30.gasv	0	–	–	0.0	0.0	–	–	–
m500-sd50-cov30.pindel	132	**87.9**	**0.0**	**61.8**	**58.1**	**72.5**	**6.8**	**10.0**
m500-sd50-cov30.prism	0	–	–	0.0	0.0	–	–	–
m500-sd50-cov30.svdetect	11	18.2	**0.0**	0.7	0.0	1.4	23.5	51.5
**Length Range 50–99** (37 true insertions)								
m500-sd50-cov30.breakdancer	0	–	–	0.0	0.0	–	–	–
m500-sd50-cov30.clever	18	**83.3**	**0.0**	**48.6**	**21.6**	**61.4**	31.4	46.3
m500-sd50-cov30.delly	0	–	–	0.0	0.0	–	–	–
m500-sd50-cov30.gasv	0	–	–	0.0	0.0	–	–	–
m500-sd50-cov30.pindel	17	58.8	**0.0**	40.5	13.5	48.0	**2.3**	**9.5**
m500-sd50-cov30.prism	0	–	–	0.0	0.0	–	–	–
m500-sd50-cov30.svdetect	29	6.9	**0.0**	13.5	5.4	9.1	12.0	39.5
**Length Range 100–249** (30 true insertions)								
m500-sd50-cov30.breakdancer	23	21.7	**0.0**	16.7	3.3	18.9	52.6	55.2
m500-sd50-cov30.clever	42	**66.7**	**0.0**	**76.7**	**60.0**	**71.3**	**38.5**	28.7
m500-sd50-cov30.delly	0	–	–	0.0	0.0	–	–	–
m500-sd50-cov30.gasv	0	–	–	0.0	0.0	–	–	–
m500-sd50-cov30.pindel	0	–	–	13.3	6.7	–	–	–
m500-sd50-cov30.prism	0	–	–	0.0	0.0	–	–	–
m500-sd50-cov30.svdetect	42	4.8	**0.0**	0.0	0.0	0.0	67.0	**9.0**
**Length Range 250–999** (19 true insertions)								
m500-sd50-cov30.breakdancer	8	**0.0**	**0.0**	0.0	0.0	–	–	–
m500-sd50-cov30.clever	0	–	–	**10.5**	**10.5**	–	–	–
m500-sd50-cov30.delly	0	–	–	0.0	0.0	–	–	–
m500-sd50-cov30.gasv	0	–	–	0.0	0.0	–	–	–
m500-sd50-cov30.pindel	0	–	–	0.0	0.0	–	–	–
m500-sd50-cov30.prism	0	–	–	0.0	0.0	–	–	–
m500-sd50-cov30.svdetect	0	–	–	0.0	0.0	–	–	–
**Length Range 1000–50000** (10 true insertions)								
m500-sd50-cov30.breakdancer	0	–	–	**0.0**	**0.0**	–	–	–
m500-sd50-cov30.clever	0	–	–	**0.0**	**0.0**	–	–	–
m500-sd50-cov30.delly	0	–	–	**0.0**	**0.0**	–	–	–
m500-sd50-cov30.gasv	0	–	–	**0.0**	**0.0**	–	–	–
m500-sd50-cov30.pindel	0	–	–	**0.0**	**0.0**	–	–	–
m500-sd50-cov30.prism	0	–	–	**0.0**	**0.0**	–	–	–
m500-sd50-cov30.svdetect	0	–	–	**0.0**	**0.0**	–	–	–

2.2 Deletions

	Abs.	Prec.	Mix.	Rec.	Exc.	F.	ΔLen.	Dist.
**Length Range 20–49** (118 true deletions)								
m500-sd50-cov30.breakdancer	0	–	–	0.0	0.0	–	–	–
m500-sd50-cov30.clever	11	**90.9**	0.0	6.8	1.7	12.6	33.3	22.9
m500-sd50-cov30.delly	0	–	–	0.0	0.0	–	–	–
m500-sd50-cov30.gasv	461	2.0	0.2	6.8	0.8	3.0	20.9	47.4
m500-sd50-cov30.pindel	74	87.8	0.0	54.2	0.0	**67.1**	**0.2**	**1.1**
m500-sd50-cov30.prism	234	54.3	**0.9**	**85.6**	**27.1**	66.4	4.6	7.1
m500-sd50-cov30.svdetect	31	12.9	0.0	3.4	0.8	5.4	52.0	41.2
**Length Range 50–99** (33 true deletions)								
m500-sd50-cov30.breakdancer	0	–	–	0.0	0.0	–	–	–
m500-sd50-cov30.clever	16	93.8	**6.2**	66.7	3.0	**77.9**	20.0	24.4
m500-sd50-cov30.delly	0	–	–	15.2	3.0	–	–	–
m500-sd50-cov30.gasv	112	6.2	0.0	3.0	0.0	4.1	33.7	40.4
m500-sd50-cov30.pindel	10	**100.0**	0.0	27.3	0.0	42.9	**4.4**	**2.5**
m500-sd50-cov30.prism	115	31.3	1.7	**72.7**	**9.1**	43.8	15.8	15.3
m500-sd50-cov30.svdetect	58	15.5	0.0	24.2	0.0	18.9	46.2	48.9
**Length Range 100–249** (19 true deletions)								
m500-sd50-cov30.breakdancer	16	37.5	0.0	31.6	5.3	34.3	50.8	62.4

m500-sd50-cov30.clever	22	**59.1**	4.5	**63.2**	**10.5**	**61.1**	27.2	34.9
m500-sd50-cov30.delly	51	25.5	0.0	36.8	0.0	30.1	49.6	24.5
m500-sd50-cov30.gasv	12	16.7	**8.3**	10.5	0.0	12.9	26.0	68.0
m500-sd50-cov30.pindel	6	50.0	0.0	21.1	0.0	29.6	**0.0**	**0.0**
m500-sd50-cov30.prism	147	8.8	0.7	52.6	**10.5**	15.1	23.6	25.1
m500-sd50-cov30.svdetect	171	5.3	0.6	42.1	0.0	9.4	49.4	31.6
**Length Range 250–999** (19 true deletions)								
m500-sd50-cov30.breakdancer	18	77.8	**0.0**	**73.7**	**0.0**	**75.7**	14.4	58.7
m500-sd50-cov30.clever	17	76.5	**0.0**	68.4	**0.0**	72.2	11.7	13.8
m500-sd50-cov30.delly	83	15.7	**0.0**	**73.7**	**0.0**	25.8	51.5	36.4
m500-sd50-cov30.gasv	3317	0.2	**0.0**	31.6	**0.0**	0.4	9.7	58.8
m500-sd50-cov30.pindel	11	**81.8**	**0.0**	47.4	**0.0**	60.0	**0.0**	**0.0**
m500-sd50-cov30.prism	40	42.5	**0.0**	63.2	**0.0**	50.8	9.9	6.9
m500-sd50-cov30.svdetect	240	3.8	**0.0**	57.9	**0.0**	7.0	46.7	25.3
**Length Range 1000–50000** (4 true deletions)								
m500-sd50-cov30.breakdancer	2	**100.0**	**0.0**	**50.0**	**0.0**	**66.7**	10.5	67.2
m500-sd50-cov30.clever	3	33.3	**0.0**	25.0	**0.0**	28.6	1.0	0.5
m500-sd50-cov30.delly	10	10.0	**0.0**	25.0	**0.0**	14.3	**0.0**	1.0
m500-sd50-cov30.gasv	5	20.0	**0.0**	25.0	**0.0**	22.2	2.0	71.0
m500-sd50-cov30.pindel	12	16.7	**0.0**	**50.0**	**0.0**	25.0	**0.0**	**0.0**
m500-sd50-cov30.prism	6	16.7	**0.0**	25.0	**0.0**	20.0	**0.0**	**0.0**
m500-sd50-cov30.svdetect	6	16.7	**0.0**	25.0	**0.0**	20.0	44.0	24.0

2.3 Table Legend

- **Abs.:** *Absolute number* of predictions made in this length range
- **Prec.:** *Precision*, the percentage of predictions in that length range that match a true deletion/insertion.
- **Mix.:** Percentage of predictions that don't match a true insertion/deletion but a *mixed insertion/deletion event* of the same/similar effective length.
- **Rec.:** *Recall*, the percentage of true insertions/deletions in that length range that have been discovered.
- **Exc.:** *Exclusive calls*: percentage of true insertions/deletions that are *only* discovered by this tool.
- **F:** *F-Measure*: $2 \cdot \text{precision} \cdot \text{recall}/(\text{precision} + \text{recall})$. This integrates precision and recall into one statistic.
- **ΔLen.:** *Length difference*: average length difference between prediction and true insertion/deletion (averaged over all predictions that match a true annotation)
- **Dist.:** *Distance*: average center distance between prediction and true insertion/deletion (averaged over all predictions that match a true annotation)
