# Supplementary material for: SV-AUTOPILOT: optimized, automated construction of structural variation discovery and benchmarking pipelines
Source: BMC Genomics. 2015 Mar 25;16(1):238. doi: 10.1186/s12864-015-1376-9 (PMC4520269; doi:10.1186/s12864-015-1376-9)
Supplement: Additional file 1: — The data sets supporting the results of this article are available in the as part of the SV-AUTOPILOT virtual machine, in https://bioimg.org/sv-autopilot . The scripts used as the basis for the virtual machine described in this article are available via the GitHub repository, in https://github.com/ALLBio/allbiotc2/. [file 12864_2015_1376_MOESM1_ESM.zip › 1993348534130930_add13.pdf]

1 Command line

```
../../allbiotc2/evaluation/evaluate-sv-predictions2 -R 20-49,50-99,100-249,250-999,1000-50000 -e
    tair9_sd15_o50z20 -o 50 -z 20 -L ../../data/reference_tair9/ler_0.v7c_reference.vcf mean500-stddev15
    -cov30.breakdancer.vcf mean500-stddev15-cov30.clever.vcf mean500-stddev15-cov30.delly.vcf mean500-
    stddev15-cov30.gasv.vcf mean500-stddev15-cov30.pindel.vcf mean500-stddev15-cov30.prism.vcf mean500-
    stddev15-cov30.svdetect.vcf
```

2 Overall performance

2.1 Insertions

	Abs.	Prec.	Mix.	Rec.	Exc.	F.	ΔLen.	Dist.
**Length Range 20–49** (8,094 true insertions)								
m500-sd15-cov30.breakdancer	0	–	–	0.0	0.0	–	–	–
m500-sd15-cov30.clever	4685	76.7	**3.1**	**30.6**	**13.3**	**43.7**	8.4	10.2
m500-sd15-cov30.delly	0	–	–	0.0	0.0	–	–	–
m500-sd15-cov30.gasv	0	–	–	0.0	0.0	–	–	–
m500-sd15-cov30.pindel	2444	**93.1**	1.0	28.4	11.2	43.5	**1.5**	**1.1**
m500-sd15-cov30.prism	0	–	–	0.0	0.0	–	–	–
m500-sd15-cov30.svdetect	0	–	–	0.0	0.0	–	–	–
**Length Range 50–99** (446 true insertions)								
m500-sd15-cov30.breakdancer	2850	0.0	0.0	0.0	0.0	0.0	17.0	35.0
m500-sd15-cov30.clever	1409	23.0	**5.9**	**60.1**	**29.8**	33.3	7.4	9.2
m500-sd15-cov30.delly	0	–	–	0.0	0.0	–	–	–
m500-sd15-cov30.gasv	0	–	–	0.0	0.0	–	–	–
m500-sd15-cov30.pindel	388	**48.2**	1.5	37.4	7.2	**42.1**	**1.9**	**2.1**
m500-sd15-cov30.prism	0	–	–	0.0	0.0	–	–	–
m500-sd15-cov30.svdetect	0	–	–	0.0	0.0	–	–	–
**Length Range 100–249** (82 true insertions)								
m500-sd15-cov30.breakdancer	310	0.0	0.0	0.0	0.0	–	–	–
m500-sd15-cov30.clever	313	**23.0**	**24.3**	**89.0**	**89.0**	**36.6**	**5.2**	**8.9**
m500-sd15-cov30.delly	0	–	–	0.0	0.0	–	–	–
m500-sd15-cov30.gasv	0	–	–	0.0	0.0	–	–	–
m500-sd15-cov30.pindel	0	–	–	0.0	0.0	–	–	–
m500-sd15-cov30.prism	0	–	–	0.0	0.0	–	–	–
m500-sd15-cov30.svdetect	0	–	–	0.0	0.0	–	–	–
**Length Range 250–999** (44 true insertions)								
m500-sd15-cov30.breakdancer	27	7.4	**25.9**	4.5	4.5	5.6	**7.5**	14.5
m500-sd15-cov30.clever	9	**55.6**	11.1	**11.4**	**11.4**	**18.9**	9.2	**3.0**
m500-sd15-cov30.delly	0	–	–	0.0	0.0	–	–	–
m500-sd15-cov30.gasv	0	–	–	0.0	0.0	–	–	–
m500-sd15-cov30.pindel	0	–	–	0.0	0.0	–	–	–
m500-sd15-cov30.prism	0	–	–	0.0	0.0	–	–	–
m500-sd15-cov30.svdetect	0	–	–	0.0	0.0	–	–	–
**Length Range 1000–50000** (3 true insertions)								
m500-sd15-cov30.breakdancer	0	–	–	**0.0**	**0.0**	–	–	–
m500-sd15-cov30.clever	0	–	–	**0.0**	**0.0**	–	–	–
m500-sd15-cov30.delly	0	–	–	**0.0**	**0.0**	–	–	–
m500-sd15-cov30.gasv	0	–	–	**0.0**	**0.0**	–	–	–
m500-sd15-cov30.pindel	0	–	–	**0.0**	**0.0**	–	–	–
m500-sd15-cov30.prism	0	–	–	**0.0**	**0.0**	–	–	–
m500-sd15-cov30.svdetect	0	–	–	**0.0**	**0.0**	–	–	–

2.2 Deletions

	Abs.	Prec.	Mix.	Rec.	Exc.	F.	ΔLen.	Dist.
**Length Range 20–49** (3,595 true deletions)								
m500-sd15-cov30.breakdancer	0	–	–	0.0	0.0	–	–	–
m500-sd15-cov30.clever	3760	74.8	**10.8**	**58.9**	**16.1**	**65.9**	6.8	9.1
m500-sd15-cov30.delly	0	–	–	0.0	0.0	–	–	–
m500-sd15-cov30.gasv	1418	20.5	1.6	7.1	0.3	10.5	7.8	35.6
m500-sd15-cov30.pindel	1684	**94.4**	4.7	43.9	5.3	59.9	**0.1**	**0.5**
m500-sd15-cov30.prism	1351	59.7	9.3	20.5	4.9	30.5	2.1	2.3
m500-sd15-cov30.svdetect	1	0.0	0.0	0.0	0.0	–	–	–
**Length Range 50–99** (781 true deletions)								
m500-sd15-cov30.breakdancer	1128	0.5	0.2	0.5	0.0	0.5	14.0	27.0
m500-sd15-cov30.clever	777	58.7	**16.2**	**62.4**	**21.6**	**60.5**	5.8	10.0
m500-sd15-cov30.delly	1	**100.0**	0.0	0.6	0.4	1.3	**0.0**	1.0
m500-sd15-cov30.gasv	287	10.5	3.8	3.7	0.5	5.5	6.5	36.6
m500-sd15-cov30.pindel	308	89.6	6.2	35.2	2.3	50.6	0.1	**0.3**
m500-sd15-cov30.prism	460	31.5	8.9	18.3	3.3	23.2	2.5	3.0
m500-sd15-cov30.svdetect	1	0.0	0.0	0.0	0.0	–	–	–
**Length Range 100–249** (393 true deletions)								
m500-sd15-cov30.breakdancer	505	4.4	3.6	5.3	0.8	4.8	7.2	28.0

m500-sd15-cov30.clever	528	48.9	**24.1**	**67.2**	**17.3**	**56.6**	6.3	10.2
m500-sd15-cov30.delly	1636	7.2	3.8	29.0	2.0	11.6	6.0	5.1
m500-sd15-cov30.gasv	188	9.0	0.5	4.6	2.5	6.1	6.5	31.4
m500-sd15-cov30.pindel	158	**89.2**	7.6	35.9	1.0	51.2	**0.0**	**0.3**
m500-sd15-cov30.prism	145	42.1	17.9	14.8	1.8	21.9	2.5	2.4
m500-sd15-cov30.svdetect	8	0.0	0.0	0.0	0.0	–	–	–
**Length Range 250–999** (572 true deletions)								
m500-sd15-cov30.breakdancer	759	17.5	9.4	23.6	1.6	20.1	7.6	30.9
m500-sd15-cov30.clever	756	54.8	**20.6**	**72.6**	3.8	**62.4**	5.8	9.2
m500-sd15-cov30.delly	1527	26.7	9.0	71.2	**5.1**	38.8	2.4	3.1
m500-sd15-cov30.gasv	5994	0.1	0.1	1.0	0.3	0.2	5.7	36.3
m500-sd15-cov30.pindel	264	**87.9**	6.1	40.6	0.7	55.5	**0.1**	**0.2**
m500-sd15-cov30.prism	275	41.8	20.0	18.7	0.9	25.8	2.0	1.8
m500-sd15-cov30.svdetect	537	5.6	5.0	5.2	0.5	5.4	9.8	10.1
**Length Range 1000–50000** (370 true deletions)								
m500-sd15-cov30.breakdancer	490	18.0	9.4	23.8	0.5	20.5	5.9	29.1
m500-sd15-cov30.clever	482	**58.1**	**19.5**	75.9	3.0	**65.8**	5.3	8.3
m500-sd15-cov30.delly	810	37.3	10.6	**81.6**	**3.5**	51.2	1.6	2.8
m500-sd15-cov30.gasv	749	0.7	0.3	1.4	0.3	0.9	11.2	40.4
m500-sd15-cov30.pindel	363	53.2	3.6	51.9	0.3	52.5	**0.0**	**1.1**
m500-sd15-cov30.prism	153	44.4	15.0	17.0	0.3	24.6	1.4	2.6
m500-sd15-cov30.svdetect	715	4.8	3.2	8.9	0.8	6.2	11.9	12.1

2.3 Table Legend

- **Abs.:** *Absolute number* of predictions made in this length range
- **Prec.:** *Precision*, the percentage of predictions in that length range that match a true deletion/insertion.
- **Mix.:** Percentage of predictions that don't match a true insertion/deletion but a *mixed insertion/deletion event* of the same/similar effective length.
- **Rec.:** *Recall*, the percentage of true insertions/deletions in that length range that have been discovered.
- **Exc.:** *Exclusive calls*: percentage of true insertions/deletions that are *only* discovered by this tool.
- **F:** *F-Measure*: $2 \cdot \text{precision} \cdot \text{recall}/(\text{precision} + \text{recall})$. This integrates precision and recall into one statistic.
- **ΔLen.:** *Length difference*: average length difference between prediction and true insertion/deletion (averaged over all predictions that match a true annotation)
- **Dist.:** *Distance*: average center distance between prediction and true insertion/deletion (averaged over all predictions that match a true annotation)
