# Supplementary material for: SV-AUTOPILOT: optimized, automated construction of structural variation discovery and benchmarking pipelines
Source: BMC Genomics. 2015 Mar 25;16(1):238. doi: 10.1186/s12864-015-1376-9 (PMC4520269; doi:10.1186/s12864-015-1376-9)
Supplement: Additional file 1: — The data sets supporting the results of this article are available in the as part of the SV-AUTOPILOT virtual machine, in https://bioimg.org/sv-autopilot . The scripts used as the basis for the virtual machine described in this article are available via the GitHub repository, in https://github.com/ALLBio/allbiotc2/. [file 12864_2015_1376_MOESM1_ESM.zip › 1993348534130930_add16.pdf]

1 Command line

```
../../allbiotc2/evaluation/evaluate-sv-predictions2 -R 20-49,50-99,100-249,250-999,1000-50000 -e
    tair9_sd15_o100z100 -o 100 -z 100 -L ../../data/reference_tair9/ler_0.v7c_reference.vcf mean500-
    stddev15-cov30.breakdancer.vcf mean500-stddev15-cov30.clever.vcf mean500-stddev15-cov30.delly.vcf
    mean500-stddev15-cov30.gasv.vcf mean500-stddev15-cov30.pindel.vcf mean500-stddev15-cov30.prism.vcf
    mean500-stddev15-cov30.svdetect.vcf
```

2 Overall performance

2.1 Insertions

	Abs.	Prec.	Mix.	Rec.	Exc.	F.	ΔLen.	Dist.
**Length Range 20–49** (8,094 true insertions)								
m500-sd15-cov30.breakdancer	0	–	–	2.5	1.0	–	–	–
m500-sd15-cov30.clever	4685	94.0	**2.5**	**56.4**	**31.5**	**70.5**	10.5	15.7
m500-sd15-cov30.delly	0	–	–	0.0	0.0	–	–	–
m500-sd15-cov30.gasv	0	–	–	0.0	0.0	–	–	–
m500-sd15-cov30.pindel	2444	**95.0**	0.9	33.8	9.3	49.8	**2.1**	**1.5**
m500-sd15-cov30.prism	0	–	–	0.0	0.0	–	–	–
m500-sd15-cov30.svdetect	0	–	–	0.0	0.0	–	–	–
**Length Range 50–99** (446 true insertions)								
m500-sd15-cov30.breakdancer	2850	19.1	0.6	0.7	0.2	1.3	51.9	52.7
m500-sd15-cov30.clever	1409	**94.3**	**5.4**	**81.2**	**43.0**	**87.3**	33.3	16.7
m500-sd15-cov30.delly	0	–	–	0.0	0.0	–	–	–
m500-sd15-cov30.gasv	0	–	–	0.0	0.0	–	–	–
m500-sd15-cov30.pindel	388	78.4	3.1	42.4	4.7	55.0	**16.8**	**13.6**
m500-sd15-cov30.prism	0	–	–	0.0	0.0	–	–	–
m500-sd15-cov30.svdetect	0	–	–	0.0	0.0	–	–	–
**Length Range 100–249** (82 true insertions)								
m500-sd15-cov30.breakdancer	310	5.5	0.3	0.0	0.0	0.0	87.8	63.5
m500-sd15-cov30.clever	313	**68.1**	**24.9**	**92.7**	**91.5**	**78.5**	**50.8**	**20.9**
m500-sd15-cov30.delly	0	–	–	0.0	0.0	–	–	–
m500-sd15-cov30.gasv	0	–	–	0.0	0.0	–	–	–
m500-sd15-cov30.pindel	0	–	–	1.2	0.0	–	–	–
m500-sd15-cov30.prism	0	–	–	0.0	0.0	–	–	–
m500-sd15-cov30.svdetect	0	–	–	0.0	0.0	–	–	–
**Length Range 250–999** (44 true insertions)								
m500-sd15-cov30.breakdancer	27	18.5	**48.1**	11.4	11.4	14.1	13.2	38.2
m500-sd15-cov30.clever	9	**66.7**	33.3	**15.9**	**15.9**	**25.7**	**11.5**	**3.7**
m500-sd15-cov30.delly	0	–	–	0.0	0.0	–	–	–
m500-sd15-cov30.gasv	0	–	–	0.0	0.0	–	–	–
m500-sd15-cov30.pindel	0	–	–	0.0	0.0	–	–	–
m500-sd15-cov30.prism	0	–	–	0.0	0.0	–	–	–
m500-sd15-cov30.svdetect	0	–	–	0.0	0.0	–	–	–
**Length Range 1000–50000** (3 true insertions)								
m500-sd15-cov30.breakdancer	0	–	–	**0.0**	**0.0**	–	–	–
m500-sd15-cov30.clever	0	–	–	**0.0**	**0.0**	–	–	–
m500-sd15-cov30.delly	0	–	–	**0.0**	**0.0**	–	–	–
m500-sd15-cov30.gasv	0	–	–	**0.0**	**0.0**	–	–	–
m500-sd15-cov30.pindel	0	–	–	**0.0**	**0.0**	–	–	–
m500-sd15-cov30.prism	0	–	–	**0.0**	**0.0**	–	–	–
m500-sd15-cov30.svdetect	0	–	–	**0.0**	**0.0**	–	–	–

2.2 Deletions

	Abs.	Prec.	Mix.	Rec.	Exc.	F.	ΔLen.	Dist.
**Length Range 20–49** (3,595 true deletions)								
m500-sd15-cov30.breakdancer	0	–	–	1.1	0.2	–	–	–
m500-sd15-cov30.clever	3760	85.5	**10.9**	**65.0**	**12.4**	**73.9**	8.5	13.5
m500-sd15-cov30.delly	0	–	–	4.3	0.1	–	–	–
m500-sd15-cov30.gasv	1418	68.3	9.4	25.1	1.0	36.8	11.1	58.6
m500-sd15-cov30.pindel	1684	94.6	4.6	44.5	3.5	60.5	**0.2**	**0.6**
m500-sd15-cov30.prism	1351	66.1	10.2	21.6	4.7	32.5	4.8	5.4
m500-sd15-cov30.svdetect	1	**100.0**	0.0	0.0	0.0	0.0	34.0	23.0
**Length Range 50–99** (781 true deletions)								
m500-sd15-cov30.breakdancer	1128	11.4	1.3	0.9	0.0	1.7	48.6	46.2
m500-sd15-cov30.clever	777	79.0	**19.0**	**82.1**	**18.3**	**80.5**	13.2	17.1
m500-sd15-cov30.delly	1	**100.0**	0.0	6.9	0.5	12.9	**0.0**	**1.0**
m500-sd15-cov30.gasv	287	76.3	16.0	35.5	1.2	48.4	15.3	60.3
m500-sd15-cov30.pindel	308	91.9	6.2	36.2	1.3	52.0	1.3	1.0
m500-sd15-cov30.prism	460	47.8	11.7	22.0	1.5	30.2	18.0	11.9
m500-sd15-cov30.svdetect	1	**100.0**	0.0	0.1	0.1	0.3	72.0	61.0
**Length Range 100–249** (393 true deletions)								
m500-sd15-cov30.breakdancer	505	10.7	8.3	9.9	0.5	10.3	20.6	37.8

m500-sd15-cov30.clever	528	64.6	**31.6**	**89.8**	**13.0**	**75.1**	12.0	17.6
m500-sd15-cov30.delly	1636	30.1	12.2	64.1	2.5	40.9	48.7	30.5
m500-sd15-cov30.gasv	188	60.1	23.9	30.8	0.0	40.7	13.4	65.8
m500-sd15-cov30.pindel	158	**89.2**	7.6	36.1	0.5	51.4	**0.0**	**0.3**
m500-sd15-cov30.prism	145	46.9	30.3	16.3	0.8	24.2	9.7	5.6
m500-sd15-cov30.svdetect	8	0.0	0.0	0.0	0.0	–	–	–
**Length Range 250–999** (572 true deletions)								
m500-sd15-cov30.breakdancer	759	64.3	24.9	87.1	**0.7**	74.0	14.0	48.3
m500-sd15-cov30.clever	756	68.4	**27.4**	91.4	0.2	**78.2**	10.9	14.0
m500-sd15-cov30.delly	1527	34.1	14.1	**93.0**	0.2	49.9	10.5	8.2
m500-sd15-cov30.gasv	5994	4.2	1.4	44.9	0.3	7.7	11.6	71.3
m500-sd15-cov30.pindel	264	**87.9**	6.4	40.6	0.3	55.5	**0.1**	**0.2**
m500-sd15-cov30.prism	275	49.5	25.8	20.3	0.0	28.8	7.8	5.2
m500-sd15-cov30.svdetect	537	65.2	25.9	58.6	0.0	61.7	43.6	25.0
**Length Range 1000–50000** (370 true deletions)								
m500-sd15-cov30.breakdancer	490	61.8	21.4	82.7	0.3	70.8	10.3	50.6
m500-sd15-cov30.clever	482	**68.3**	**24.5**	90.0	0.0	**77.6**	9.5	13.4
m500-sd15-cov30.delly	810	43.3	14.9	95.4	0.0	59.6	6.7	5.6
m500-sd15-cov30.gasv	749	25.8	6.4	52.4	0.0	34.6	11.0	74.2
m500-sd15-cov30.pindel	363	54.0	3.9	52.2	0.0	53.1	**0.4**	**1.9**
m500-sd15-cov30.prism	153	47.7	18.3	17.3	0.0	25.4	5.3	4.5
m500-sd15-cov30.svdetect	715	48.5	16.4	**97.0**	**0.5**	64.7	42.7	23.7

2.3 Table Legend

- **Abs.:** *Absolute number* of predictions made in this length range
- **Prec.:** *Precision*, the percentage of predictions in that length range that match a true deletion/insertion.
- **Mix.:** Percentage of predictions that don't match a true insertion/deletion but a *mixed insertion/deletion event* of the same/similar effective length.
- **Rec.:** *Recall*, the percentage of true insertions/deletions in that length range that have been discovered.
- **Exc.:** *Exclusive calls*: percentage of true insertions/deletions that are *only* discovered by this tool.
- **F:** *F-Measure*: $2 \cdot \text{precision} \cdot \text{recall}/(\text{precision} + \text{recall})$. This integrates precision and recall into one statistic.
- **ΔLen.:** *Length difference*: average length difference between prediction and true insertion/deletion (averaged over all predictions that match a true annotation)
- **Dist.:** *Distance*: average center distance between prediction and true insertion/deletion (averaged over all predictions that match a true annotation)
