# Supplementary material for: SV-AUTOPILOT: optimized, automated construction of structural variation discovery and benchmarking pipelines
Source: BMC Genomics. 2015 Mar 25;16(1):238. doi: 10.1186/s12864-015-1376-9 (PMC4520269; doi:10.1186/s12864-015-1376-9)
Supplement: Additional file 1: — The data sets supporting the results of this article are available in the as part of the SV-AUTOPILOT virtual machine, in https://bioimg.org/sv-autopilot . The scripts used as the basis for the virtual machine described in this article are available via the GitHub repository, in https://github.com/ALLBio/allbiotc2/. [file 12864_2015_1376_MOESM1_ESM.zip › 1993348534130930_add19.pdf]

1 Command line

```
../../allbiotc2/evaluation/evaluate-sv-predictions2 -R 20-49,50-99,100-249,250-999,1000-50000 -e
    tair9_sd50_o50z20 -o 50 -z 20 -L ../../data/reference_tair9/ler_0.v7c_reference.vcf mean500-stddev50
    -cov30.breakdancer.vcf mean500-stddev50-cov30.clever.vcf mean500-stddev50-cov30.delly.vcf mean500-
    stddev50-cov30.gasv.vcf mean500-stddev50-cov30.pindel.vcf mean500-stddev50-cov30.prism.vcf mean500-
    stddev50-cov30.svdetect.vcf
```

2 Overall performance

2.1 Insertions

	Abs.	Prec.	Mix.	Rec.	Exc.	F.	ΔLen.	Dist.
**Length Range 20–49** (8,094 true insertions)								
m500-sd50-cov30.breakdancer	0	–	–	0.0	0.0	–	–	–
m500-sd50-cov30.clever	0	–	–	0.0	0.0	–	–	–
m500-sd50-cov30.delly	0	–	–	0.0	0.0	–	–	–
m500-sd50-cov30.gasv	0	–	–	0.0	0.0	–	–	–
m500-sd50-cov30.pindel	2510	**92.6**	**0.8**	**29.0**	**29.0**	**44.2**	**1.6**	**1.0**
m500-sd50-cov30.prism	0	–	–	0.0	0.0	–	–	–
m500-sd50-cov30.svdetect	0	–	–	0.0	0.0	–	–	–
**Length Range 50–99** (446 true insertions)								
m500-sd50-cov30.breakdancer	0	–	–	0.0	0.0	–	–	–
m500-sd50-cov30.clever	371	12.4	**4.6**	10.3	9.4	11.3	8.7	8.3
m500-sd50-cov30.delly	0	–	–	0.0	0.0	–	–	–
m500-sd50-cov30.gasv	0	–	–	0.0	0.0	–	–	–
m500-sd50-cov30.pindel	418	**45.9**	1.4	**38.1**	**37.2**	**41.7**	**2.1**	**2.1**
m500-sd50-cov30.prism	0	–	–	0.0	0.0	–	–	–
m500-sd50-cov30.svdetect	0	–	–	0.0	0.0	–	–	–
**Length Range 100–249** (82 true insertions)								
m500-sd50-cov30.breakdancer	580	0.0	0.0	0.0	0.0	–	–	–
m500-sd50-cov30.clever	506	**8.3**	**9.5**	**51.2**	**51.2**	**14.3**	**8.7**	**10.0**
m500-sd50-cov30.delly	0	–	–	0.0	0.0	–	–	–
m500-sd50-cov30.gasv	0	–	–	0.0	0.0	–	–	–
m500-sd50-cov30.pindel	0	–	–	0.0	0.0	–	–	–
m500-sd50-cov30.prism	0	–	–	0.0	0.0	–	–	–
m500-sd50-cov30.svdetect	0	–	–	0.0	0.0	–	–	–
**Length Range 250–999** (44 true insertions)								
m500-sd50-cov30.breakdancer	48	4.2	8.3	**4.5**	**4.5**	4.3	**10.5**	38.0
m500-sd50-cov30.clever	10	**20.0**	**10.0**	**4.5**	**4.5**	**7.4**	19.5	**2.0**
m500-sd50-cov30.delly	0	–	–	0.0	0.0	–	–	–
m500-sd50-cov30.gasv	0	–	–	0.0	0.0	–	–	–
m500-sd50-cov30.pindel	0	–	–	0.0	0.0	–	–	–
m500-sd50-cov30.prism	0	–	–	0.0	0.0	–	–	–
m500-sd50-cov30.svdetect	0	–	–	0.0	0.0	–	–	–
**Length Range 1000–50000** (3 true insertions)								
m500-sd50-cov30.breakdancer	0	–	–	**0.0**	**0.0**	–	–	–
m500-sd50-cov30.clever	0	–	–	**0.0**	**0.0**	–	–	–
m500-sd50-cov30.delly	0	–	–	**0.0**	**0.0**	–	–	–
m500-sd50-cov30.gasv	0	–	–	**0.0**	**0.0**	–	–	–
m500-sd50-cov30.pindel	0	–	–	**0.0**	**0.0**	–	–	–
m500-sd50-cov30.prism	0	–	–	**0.0**	**0.0**	–	–	–
m500-sd50-cov30.svdetect	0	–	–	**0.0**	**0.0**	–	–	–

2.2 Deletions

	Abs.	Prec.	Mix.	Rec.	Exc.	F.	ΔLen.	Dist.
**Length Range 20–49** (3,595 true deletions)								
m500-sd50-cov30.breakdancer	0	–	–	0.0	0.0	–	–	–
m500-sd50-cov30.clever	98	28.6	3.1	1.4	0.4	2.7	8.0	8.6
m500-sd50-cov30.delly	0	–	–	0.0	0.0	–	–	–
m500-sd50-cov30.gasv	1275	2.6	0.4	0.7	0.3	1.1	9.8	34.0
m500-sd50-cov30.pindel	1680	**94.3**	4.8	**43.8**	**32.8**	**59.8**	**0.1**	**0.5**
m500-sd50-cov30.prism	1329	61.3	**9.3**	20.8	10.4	31.0	2.1	2.2
m500-sd50-cov30.svdetect	2	0.0	0.0	0.0	0.0	–	–	–
**Length Range 50–99** (781 true deletions)								
m500-sd50-cov30.breakdancer	0	–	–	0.0	0.0	–	–	–
m500-sd50-cov30.clever	598	48.5	**10.7**	35.2	10.8	40.8	9.6	7.6
m500-sd50-cov30.delly	1	**100.0**	0.0	0.6	0.1	1.3	**0.0**	1.0
m500-sd50-cov30.gasv	583	0.9	0.7	0.9	0.5	0.9	13.0	34.9
m500-sd50-cov30.pindel	311	90.7	5.8	**35.6**	**11.4**	**51.1**	0.1	**0.3**
m500-sd50-cov30.prism	631	22.2	6.2	17.8	6.5	19.8	2.7	2.8
m500-sd50-cov30.svdetect	6	0.0	0.0	0.0	0.0	–	–	–
**Length Range 100–249** (393 true deletions)								
m500-sd50-cov30.breakdancer	335	3.0	1.5	3.3	0.3	3.1	8.0	14.1

m500-sd50-cov30.clever	650	39.8	**16.5**	**63.4**	**20.1**	48.9	8.7	8.8
m500-sd50-cov30.delly	711	8.0	4.9	13.2	2.8	10.0	9.1	9.1
m500-sd50-cov30.gasv	231	6.9	3.9	3.8	1.5	4.9	7.5	36.2
m500-sd50-cov30.pindel	163	**86.5**	7.4	35.9	3.3	**50.7**	**0.0**	**0.3**
m500-sd50-cov30.prism	758	11.6	4.5	20.6	4.1	14.9	2.8	2.5
m500-sd50-cov30.svdetect	9	0.0	0.0	0.0	0.0	–	–	–
**Length Range 250–999** (572 true deletions)								
m500-sd50-cov30.breakdancer	850	18.6	10.0	27.1	2.8	22.1	8.8	30.9
m500-sd50-cov30.clever	794	51.3	**18.6**	**71.3**	**10.5**	**59.7**	8.1	7.5
m500-sd50-cov30.delly	1114	21.5	7.9	42.0	2.1	28.4	2.5	2.9
m500-sd50-cov30.gasv	9354	0.1	0.1	2.1	0.2	0.2	11.1	44.7
m500-sd50-cov30.pindel	263	**89.4**	5.3	41.1	1.4	56.3	**0.1**	**0.2**
m500-sd50-cov30.prism	340	34.4	14.7	19.4	0.7	24.8	2.0	1.5
m500-sd50-cov30.svdetect	615	6.7	5.0	7.2	0.5	6.9	12.5	12.9
**Length Range 1000–50000** (370 true deletions)								
m500-sd50-cov30.breakdancer	520	17.7	9.6	24.9	2.4	20.7	8.6	29.8
m500-sd50-cov30.clever	497	51.9	**15.5**	70.3	**3.8**	**59.7**	7.5	7.4
m500-sd50-cov30.delly	721	40.8	11.2	**79.5**	3.5	53.9	1.4	2.7
m500-sd50-cov30.gasv	711	0.6	0.3	1.1	0.3	0.7	6.8	42.1
m500-sd50-cov30.pindel	329	**61.1**	4.0	54.1	0.0	57.4	**0.0**	**1.0**
m500-sd50-cov30.prism	136	51.5	15.4	17.3	0.5	25.9	1.7	2.8
m500-sd50-cov30.svdetect	636	4.9	3.6	8.4	0.5	6.2	12.7	12.4

2.3 Table Legend

- **Abs.:** *Absolute number* of predictions made in this length range
- **Prec.:** *Precision*, the percentage of predictions in that length range that match a true deletion/insertion.
- **Mix.:** Percentage of predictions that don't match a true insertion/deletion but a *mixed insertion/deletion event* of the same/similar effective length.
- **Rec.:** *Recall*, the percentage of true insertions/deletions in that length range that have been discovered.
- **Exc.:** *Exclusive calls*: percentage of true insertions/deletions that are *only* discovered by this tool.
- **F:** *F-Measure*: $2 \cdot \text{precision} \cdot \text{recall}/(\text{precision} + \text{recall})$. This integrates precision and recall into one statistic.
- **ΔLen.:** *Length difference*: average length difference between prediction and true insertion/deletion (averaged over all predictions that match a true annotation)
- **Dist.:** *Distance*: average center distance between prediction and true insertion/deletion (averaged over all predictions that match a true annotation)
