# Supplementary material for: SV-AUTOPILOT: optimized, automated construction of structural variation discovery and benchmarking pipelines
Source: BMC Genomics. 2015 Mar 25;16(1):238. doi: 10.1186/s12864-015-1376-9 (PMC4520269; doi:10.1186/s12864-015-1376-9)
Supplement: Additional file 1: — The data sets supporting the results of this article are available in the as part of the SV-AUTOPILOT virtual machine, in https://bioimg.org/sv-autopilot . The scripts used as the basis for the virtual machine described in this article are available via the GitHub repository, in https://github.com/ALLBio/allbiotc2/. [file 12864_2015_1376_MOESM1_ESM.zip › 1993348534130930_add22.pdf]

1 Command line

```
../../allbiotc2/evaluation/evaluate-sv-predictions2 -R 20-49,50-99,100-249,250-999,1000-50000 -e
    tair9_sd50_o100z100 -o 100 -z 100 -L ../../data/reference_tair9/ler_0.v7c_reference.vcf mean500-
    stddev50-cov30.breakdancer.vcf mean500-stddev50-cov30.clever.vcf mean500-stddev50-cov30.delly.vcf
    mean500-stddev50-cov30.gasv.vcf mean500-stddev50-cov30.pindel.vcf mean500-stddev50-cov30.prism.vcf
    mean500-stddev50-cov30.svdetect.vcf
```

2 Overall performance

2.1 Insertions

	Abs.	Prec.	Mix.	Rec.	Exc.	F.	ΔLen.	Dist.
**Length Range 20–49** (8,094 true insertions)								
m500-sd50-cov30.breakdancer	0	–	–	0.0	0.0	–	–	–
m500-sd50-cov30.clever	0	–	–	11.4	8.6	–	–	–
m500-sd50-cov30.delly	0	–	–	0.0	0.0	–	–	–
m500-sd50-cov30.gasv	0	–	–	0.0	0.0	–	–	–
m500-sd50-cov30.pindel	2510	**94.9**	**0.7**	**34.6**	**31.8**	**50.7**	**2.1**	**1.8**
m500-sd50-cov30.prism	0	–	–	0.0	0.0	–	–	–
m500-sd50-cov30.svdetect	0	–	–	0.0	0.0	–	–	–
**Length Range 50–99** (446 true insertions)								
m500-sd50-cov30.breakdancer	0	–	–	0.0	0.0	–	–	–
m500-sd50-cov30.clever	371	**94.6**	**4.6**	35.4	27.1	51.5	50.3	19.3
m500-sd50-cov30.delly	0	–	–	0.0	0.0	–	–	–
m500-sd50-cov30.gasv	0	–	–	0.0	0.0	–	–	–
m500-sd50-cov30.pindel	418	74.9	3.3	**43.0**	**34.8**	**54.7**	**17.6**	**14.2**
m500-sd50-cov30.prism	0	–	–	0.0	0.0	–	–	–
m500-sd50-cov30.svdetect	0	–	–	0.0	0.0	–	–	–
**Length Range 100–249** (82 true insertions)								
m500-sd50-cov30.breakdancer	580	1.4	3.6	9.8	0.0	2.4	65.9	64.2
m500-sd50-cov30.clever	506	**73.9**	**18.2**	**95.1**	**84.1**	**83.2**	**61.6**	**20.9**
m500-sd50-cov30.delly	0	–	–	0.0	0.0	–	–	–
m500-sd50-cov30.gasv	0	–	–	0.0	0.0	–	–	–
m500-sd50-cov30.pindel	0	–	–	1.2	0.0	–	–	–
m500-sd50-cov30.prism	0	–	–	0.0	0.0	–	–	–
m500-sd50-cov30.svdetect	0	–	–	0.0	0.0	–	–	–
**Length Range 250–999** (44 true insertions)								
m500-sd50-cov30.breakdancer	48	29.2	**35.4**	**31.8**	**29.5**	30.4	34.4	39.0
m500-sd50-cov30.clever	10	**60.0**	30.0	25.0	22.7	**35.3**	**32.8**	**4.3**
m500-sd50-cov30.delly	0	–	–	0.0	0.0	–	–	–
m500-sd50-cov30.gasv	0	–	–	0.0	0.0	–	–	–
m500-sd50-cov30.pindel	0	–	–	0.0	0.0	–	–	–
m500-sd50-cov30.prism	0	–	–	0.0	0.0	–	–	–
m500-sd50-cov30.svdetect	0	–	–	0.0	0.0	–	–	–
**Length Range 1000–50000** (3 true insertions)								
m500-sd50-cov30.breakdancer	0	–	–	**0.0**	**0.0**	–	–	–
m500-sd50-cov30.clever	0	–	–	**0.0**	**0.0**	–	–	–
m500-sd50-cov30.delly	0	–	–	**0.0**	**0.0**	–	–	–
m500-sd50-cov30.gasv	0	–	–	**0.0**	**0.0**	–	–	–
m500-sd50-cov30.pindel	0	–	–	**0.0**	**0.0**	–	–	–
m500-sd50-cov30.prism	0	–	–	**0.0**	**0.0**	–	–	–
m500-sd50-cov30.svdetect	0	–	–	**0.0**	**0.0**	–	–	–

2.2 Deletions

	Abs.	Prec.	Mix.	Rec.	Exc.	F.	ΔLen.	Dist.
**Length Range 20–49** (3,595 true deletions)								
m500-sd50-cov30.breakdancer	0	–	–	0.0	0.0	–	–	–
m500-sd50-cov30.clever	98	67.3	**11.2**	4.5	1.4	8.4	20.5	26.3
m500-sd50-cov30.delly	0	–	–	0.2	0.1	–	–	–
m500-sd50-cov30.gasv	1275	14.9	2.1	4.1	1.3	6.5	18.2	63.5
m500-sd50-cov30.pindel	1680	**94.5**	4.8	**44.5**	**31.1**	**60.5**	**0.1**	**0.6**
m500-sd50-cov30.prism	1329	67.4	9.7	22.0	10.7	33.1	4.5	5.1
m500-sd50-cov30.svdetect	2	50.0	0.0	0.0	0.0	0.0	43.0	36.5
**Length Range 50–99** (781 true deletions)								
m500-sd50-cov30.breakdancer	0	–	–	0.0	0.0	–	–	–
m500-sd50-cov30.clever	598	81.1	**16.7**	**52.9**	**13.8**	**64.0**	22.3	14.5
m500-sd50-cov30.delly	1	**100.0**	0.0	5.9	0.4	11.1	**0.0**	1.0
m500-sd50-cov30.gasv	583	29.7	6.9	12.3	1.5	17.4	33.1	65.2
m500-sd50-cov30.pindel	311	92.3	5.8	36.6	4.7	52.4	0.8	**0.7**
m500-sd50-cov30.prism	631	37.7	8.7	22.0	7.4	27.8	22.5	13.7
m500-sd50-cov30.svdetect	6	16.7	0.0	0.1	0.1	0.3	52.0	5.0
**Length Range 100–249** (393 true deletions)								
m500-sd50-cov30.breakdancer	335	19.4	10.1	16.8	1.0	18.0	48.2	35.4

m500-sd50-cov30.clever	650	63.7	**32.5**	**91.9**	**9.2**	**75.2**	22.2	14.3
m500-sd50-cov30.delly	711	55.1	24.6	69.0	1.0	61.3	43.9	26.3
m500-sd50-cov30.gasv	231	54.5	26.8	29.5	0.0	38.3	28.2	61.2
m500-sd50-cov30.pindel	163	**86.5**	7.4	36.1	0.0	51.0	**0.0**	**0.3**
m500-sd50-cov30.prism	758	16.2	7.8	23.7	1.0	19.3	19.7	13.2
m500-sd50-cov30.svdetect	9	11.1	0.0	0.3	0.3	0.5	40.0	20.0
**Length Range 250–999** (572 true deletions)								
m500-sd50-cov30.breakdancer	850	60.7	25.8	89.7	**0.5**	72.4	15.1	48.6
m500-sd50-cov30.clever	794	67.5	**29.0**	94.1	0.3	**78.6**	14.0	11.0
m500-sd50-cov30.delly	1114	43.2	18.6	**94.9**	0.2	59.4	25.9	14.6
m500-sd50-cov30.gasv	9354	2.6	0.8	44.6	0.2	5.0	23.5	72.8
m500-sd50-cov30.pindel	263	**89.4**	5.3	41.1	0.3	56.3	**0.1**	**0.2**
m500-sd50-cov30.prism	340	41.2	17.9	21.7	0.2	28.4	7.7	5.3
m500-sd50-cov30.svdetect	615	62.6	23.9	64.2	0.2	63.4	41.5	24.2
**Length Range 1000–50000** (370 true deletions)								
m500-sd50-cov30.breakdancer	520	62.7	21.7	88.6	0.3	73.4	14.3	51.2
m500-sd50-cov30.clever	497	**69.6**	**25.4**	94.3	0.0	**80.1**	14.7	11.0
m500-sd50-cov30.delly	721	47.9	17.3	94.6	0.0	63.6	7.9	5.8
m500-sd50-cov30.gasv	711	25.6	7.7	50.3	0.0	33.9	20.5	74.6
m500-sd50-cov30.pindel	329	61.7	4.0	54.3	0.0	57.8	**0.0**	**1.6**
m500-sd50-cov30.prism	136	57.4	19.1	17.8	0.0	27.2	6.0	6.3
m500-sd50-cov30.svdetect	636	53.8	18.7	**96.2**	**0.5**	69.0	42.9	23.7

2.3 Table Legend

- **Abs.:** *Absolute number* of predictions made in this length range
- **Prec.:** *Precision*, the percentage of predictions in that length range that match a true deletion/insertion.
- **Mix.:** Percentage of predictions that don't match a true insertion/deletion but a *mixed insertion/deletion event* of the same/similar effective length.
- **Rec.:** *Recall*, the percentage of true insertions/deletions in that length range that have been discovered.
- **Exc.:** *Exclusive calls*: percentage of true insertions/deletions that are *only* discovered by this tool.
- **F:** *F-Measure*: $2 \cdot \text{precision} \cdot \text{recall}/(\text{precision} + \text{recall})$. This integrates precision and recall into one statistic.
- **ΔLen.:** *Length difference*: average length difference between prediction and true insertion/deletion (averaged over all predictions that match a true annotation)
- **Dist.:** *Distance*: average center distance between prediction and true insertion/deletion (averaged over all predictions that match a true annotation)
