# Supplementary material for: SV-AUTOPILOT: optimized, automated construction of structural variation discovery and benchmarking pipelines
Source: BMC Genomics. 2015 Mar 25;16(1):238. doi: 10.1186/s12864-015-1376-9 (PMC4520269; doi:10.1186/s12864-015-1376-9)
Supplement: Additional file 1: — The data sets supporting the results of this article are available in the as part of the SV-AUTOPILOT virtual machine, in https://bioimg.org/sv-autopilot . The scripts used as the basis for the virtual machine described in this article are available via the GitHub repository, in https://github.com/ALLBio/allbiotc2/. [file 12864_2015_1376_MOESM1_ESM.zip › 1993348534130930_add25.pdf]

1 Command line

```
../../allbiotc2/evaluation/evaluate-sv-predictions2 -R 20-49,50-99,100-249,250-999,1000-50000 -L -o 50 -z
     20 ../../allbio/data/reference_human/venter.phased.b37.nodots.vcf venter-sim.500_15.breakdancer.vcf
     venter-sim.500_15.clever.vcf venter-sim.500_15.delly.vcf venter-sim.500_15.gasv.vcf venter-sim.500
    _15.pindel.vcf venter-sim.500_15.svdetect.vcf
```

2 Overall performance

2.1 Insertions

	Abs.	Prec.	Mix.	Rec.	Exc.	F.	ΔLen.	Dist.
**Length Range 20–49** (8,641 true insertions)								
venter-sim.500-15.breakdancer	0	–	–	0.0	0.0	–	–	–
venter-sim.500-15.clever	9090	**83.6**	0.0	**61.1**	**16.2**	**70.6**	6.3	15.9
venter-sim.500-15.delly	0	–	–	0.0	0.0	–	–	–
venter-sim.500-15.gasv	0	–	–	0.0	0.0	–	–	–
venter-sim.500-15.pindel	7117	79.9	**0.1**	58.7	13.9	67.7	**0.9**	**3.1**
venter-sim.500-15.svdetect	0	–	–	0.0	0.0	–	–	–
**Length Range 50–99** (1,964 true insertions)								
venter-sim.500-15.breakdancer	4170	0.4	**0.0**	0.7	0.7	0.5	9.2	24.9
venter-sim.500-15.clever	1420	**67.4**	**0.0**	**47.8**	**35.9**	**55.9**	4.5	17.1
venter-sim.500-15.delly	0	–	–	0.0	0.0	–	–	–
venter-sim.500-15.gasv	0	–	–	0.0	0.0	–	–	–
venter-sim.500-15.pindel	704	48.9	**0.0**	16.1	4.2	24.2	**1.2**	**7.8**
venter-sim.500-15.svdetect	0	–	–	0.0	0.0	–	–	–
**Length Range 100–249** (1,371 true insertions)								
venter-sim.500-15.breakdancer	1172	0.2	**0.0**	0.2	0.1	0.2	9.0	28.5
venter-sim.500-15.clever	929	**61.2**	**0.0**	**41.1**	**41.0**	**49.2**	**4.8**	**15.9**
venter-sim.500-15.delly	0	–	–	0.0	0.0	–	–	–
venter-sim.500-15.gasv	0	–	–	0.0	0.0	–	–	–
venter-sim.500-15.pindel	0	–	–	0.0	0.0	–	–	–
venter-sim.500-15.svdetect	0	–	–	0.0	0.0	–	–	–
**Length Range 250–999** (1,281 true insertions)								
venter-sim.500-15.breakdancer	549	0.9	**0.0**	0.4	0.4	0.5	**7.4**	20.2
venter-sim.500-15.clever	342	**17.0**	**0.0**	**4.8**	**4.8**	**7.5**	8.4	**10.5**
venter-sim.500-15.delly	0	–	–	0.0	0.0	–	–	–
venter-sim.500-15.gasv	0	–	–	0.0	0.0	–	–	–
venter-sim.500-15.pindel	0	–	–	0.0	0.0	–	–	–
venter-sim.500-15.svdetect	0	–	–	0.0	0.0	–	–	–
**Length Range 1000–50000** (313 true insertions)								
venter-sim.500-15.breakdancer	0	–	–	**0.0**	**0.0**	–	–	–
venter-sim.500-15.clever	0	–	–	**0.0**	**0.0**	–	–	–
venter-sim.500-15.delly	0	–	–	**0.0**	**0.0**	–	–	–
venter-sim.500-15.gasv	0	–	–	**0.0**	**0.0**	–	–	–
venter-sim.500-15.pindel	0	–	–	**0.0**	**0.0**	–	–	–
venter-sim.500-15.svdetect	0	–	–	**0.0**	**0.0**	–	–	–

2.2 Deletions

	Abs.	Prec.	Mix.	Rec.	Exc.	F.	ΔLen.	Dist.
**Length Range 20–49** (8,341 true deletions)								
venter-sim.500-15.breakdancer	0	–	–	0.1	0.1	–	–	–
venter-sim.500-15.clever	8034	86.2	**0.1**	**69.8**	**14.7**	**77.2**	6.8	14.4
venter-sim.500-15.delly	0	–	–	0.0	0.0	–	–	–
venter-sim.500-15.gasv	4686	27.2	0.0	14.2	1.5	18.7	6.6	33.9
venter-sim.500-15.pindel	5336	**93.4**	0.0	59.2	6.5	72.4	**0.3**	**0.9**
venter-sim.500-15.svdetect	2	0.0	0.0	0.0	0.0	–	–	–
**Length Range 50–99** (1,784 true deletions)								
venter-sim.500-15.breakdancer	4979	0.5	0.0	0.8	0.7	0.6	9.5	26.3
venter-sim.500-15.clever	1157	71.2	0.0	**53.4**	**25.7**	**61.0**	6.3	16.9
venter-sim.500-15.delly	1	0.0	0.0	0.6	0.2	0.0	–	–
venter-sim.500-15.gasv	518	37.3	0.0	11.2	3.6	17.2	6.3	30.6
venter-sim.500-15.pindel	614	**75.9**	**0.2**	26.0	3.5	38.7	**0.2**	**0.8**
venter-sim.500-15.svdetect	7	0.0	0.0	0.0	0.0	–	–	–
**Length Range 100–249** (1,122 true deletions)								
venter-sim.500-15.breakdancer	875	8.6	**0.0**	7.0	3.3	7.7	4.3	24.4
venter-sim.500-15.clever	750	58.1	**0.0**	**40.0**	**13.5**	**47.4**	5.7	15.5
venter-sim.500-15.delly	5422	3.0	**0.0**	14.1	2.9	5.0	7.6	10.8
venter-sim.500-15.gasv	449	16.5	**0.0**	7.1	4.2	10.0	7.5	35.1
venter-sim.500-15.pindel	375	**63.7**	**0.0**	21.2	2.0	31.8	**0.1**	**0.8**
venter-sim.500-15.svdetect	53	0.0	**0.0**	0.0	0.0	–	–	–
**Length Range 250–999** (1,504 true deletions)								
venter-sim.500-15.breakdancer	1202	20.7	**0.1**	16.4	1.7	18.3	2.9	26.8
venter-sim.500-15.clever	1151	81.1	0.0	**62.1**	**2.7**	**70.3**	5.1	9.9

venter-sim.500-15.delly	5297	13.6	0.0	47.7	1.3	21.2	0.8	2.6
venter-sim.500-15.gasv	116460	0.1	0.0	4.0	2.1	0.1	7.1	33.7
venter-sim.500-15.pindel	920	**88.2**	0.0	53.9	1.3	66.9	**0.0**	**0.1**
venter-sim.500-15.svdetect	176	2.8	0.0	0.3	0.0	0.6	10.0	30.8
**Length Range 1000–50000** (299 true deletions)								
venter-sim.500-15.breakdancer	291	16.2	**0.0**	16.1	1.3	16.1	3.0	28.9
venter-sim.500-15.clever	210	**80.5**	**0.0**	57.2	1.3	**66.9**	4.6	10.5
venter-sim.500-15.delly	872	20.4	**0.0**	**59.9**	**2.7**	30.4	0.6	2.5
venter-sim.500-15.gasv	471	1.3	**0.0**	2.0	1.0	1.6	6.8	29.8
venter-sim.500-15.pindel	459	35.9	**0.0**	54.2	0.7	43.2	**0.0**	**1.0**
venter-sim.500-15.svdetect	83	1.2	**0.0**	0.3	0.0	0.5	20.0	37.0

2.3 Table Legend

- **Abs.:** *Absolute number* of predictions made in this length range
- **Prec.:** *Precision*, the percentage of predictions in that length range that match a true deletion/insertion.
- **Mix.:** Percentage of predictions that don't match a true insertion/deletion but a *mixed insertion/deletion event* of the same/similar effective length.
- **Rec.:** *Recall*, the percentage of true insertions/deletions in that length range that have been discovered.
- **Exc.:** *Exclusive calls*: percentage of true insertions/deletions that are *only* discovered by this tool.
- **F:** *F-Measure*: $2 \cdot \text{precision} \cdot \text{recall}/(\text{precision} + \text{recall})$. This integrates precision and recall into one statistic.
- **ΔLen.:** *Length difference*: average length difference between prediction and true insertion/deletion (averaged over all predictions that match a true annotation)
- **Dist.:** *Distance*: average center distance between prediction and true insertion/deletion (averaged over all predictions that match a true annotation)
