# Supplementary material for: SV-AUTOPILOT: optimized, automated construction of structural variation discovery and benchmarking pipelines
Source: BMC Genomics. 2015 Mar 25;16(1):238. doi: 10.1186/s12864-015-1376-9 (PMC4520269; doi:10.1186/s12864-015-1376-9)
Supplement: Additional file 1: — The data sets supporting the results of this article are available in the as part of the SV-AUTOPILOT virtual machine, in https://bioimg.org/sv-autopilot . The scripts used as the basis for the virtual machine described in this article are available via the GitHub repository, in https://github.com/ALLBio/allbiotc2/. [file 12864_2015_1376_MOESM1_ESM.zip › 1993348534130930_add26.pdf]

1 Command line

```
../../allbiotc2/evaluation/evaluate-sv-predictions2 -R 20-49,50-99,100-249,250-999,1000-50000 -L -o 100 -
    z 100 ../../allbio/data/reference_human/venter.phased.b37.nodots.vcf venter-sim.500_15.breakdancer.
    vcf venter-sim.500_15.clever.vcf venter-sim.500_15.delly.vcf venter-sim.500_15.gasv.vcf venter-sim
    .500_15.pindel.vcf venter-sim.500_15.svdetect.vcf
```

2 Overall performance

2.1 Insertions

	Abs.	Prec.	Mix.	Rec.	Exc.	F.	ΔLen.	Dist.
**Length Range 20–49** (8,641 true insertions)								
venter-sim.500-15.breakdancer	0	–	–	2.6	0.3	–	–	–
venter-sim.500-15.clever	9090	**96.2**	0.0	**72.5**	**18.4**	**82.7**	6.7	22.1
venter-sim.500-15.delly	0	–	–	0.0	0.0	–	–	–
venter-sim.500-15.gasv	0	–	–	0.0	0.0	–	–	–
venter-sim.500-15.pindel	7117	88.5	**0.1**	61.5	7.9	72.6	**4.5**	**5.2**
venter-sim.500-15.svdetect	0	–	–	0.0	0.0	–	–	–
**Length Range 50–99** (1,964 true insertions)								
venter-sim.500-15.breakdancer	4170	7.2	0.1	1.7	1.2	2.8	37.0	47.3
venter-sim.500-15.clever	1420	**93.2**	0.0	**68.2**	**39.5**	**78.7**	**7.9**	27.2
venter-sim.500-15.delly	0	–	–	0.0	0.0	–	–	–
venter-sim.500-15.gasv	0	–	–	0.0	0.0	–	–	–
venter-sim.500-15.pindel	704	62.2	**0.3**	33.4	4.9	43.5	10.1	**14.0**
venter-sim.500-15.svdetect	0	–	–	0.0	0.0	–	–	–
**Length Range 100–249** (1,371 true insertions)								
venter-sim.500-15.breakdancer	1172	1.3	**0.0**	0.9	0.7	1.0	42.1	56.9
venter-sim.500-15.clever	929	**92.7**	**0.0**	**52.4**	**46.0**	**66.9**	**17.4**	**25.0**
venter-sim.500-15.delly	0	–	–	0.0	0.0	–	–	–
venter-sim.500-15.gasv	0	–	–	0.0	0.0	–	–	–
venter-sim.500-15.pindel	0	–	–	8.5	2.2	–	–	–
venter-sim.500-15.svdetect	0	–	–	0.0	0.0	–	–	–
**Length Range 250–999** (1,281 true insertions)								
venter-sim.500-15.breakdancer	549	1.8	**0.0**	0.9	0.9	1.2	**15.1**	44.1
venter-sim.500-15.clever	342	**98.2**	**0.0**	**33.0**	**33.0**	**49.4**	45.0	**17.4**
venter-sim.500-15.delly	0	–	–	0.0	0.0	–	–	–
venter-sim.500-15.gasv	0	–	–	0.0	0.0	–	–	–
venter-sim.500-15.pindel	0	–	–	0.0	0.0	–	–	–
venter-sim.500-15.svdetect	0	–	–	0.0	0.0	–	–	–
**Length Range 1000–50000** (313 true insertions)								
venter-sim.500-15.breakdancer	0	–	–	**0.0**	**0.0**	–	–	–
venter-sim.500-15.clever	0	–	–	**0.0**	**0.0**	–	–	–
venter-sim.500-15.delly	0	–	–	**0.0**	**0.0**	–	–	–
venter-sim.500-15.gasv	0	–	–	**0.0**	**0.0**	–	–	–
venter-sim.500-15.pindel	0	–	–	**0.0**	**0.0**	–	–	–
venter-sim.500-15.svdetect	0	–	–	**0.0**	**0.0**	–	–	–

2.2 Deletions

	Abs.	Prec.	Mix.	Rec.	Exc.	F.	ΔLen.	Dist.
**Length Range 20–49** (8,341 true deletions)								
venter-sim.500-15.breakdancer	0	–	–	5.8	0.4	–	–	–
venter-sim.500-15.clever	8034	**96.2**	0.0	**78.0**	**13.0**	**86.2**	7.3	19.1
venter-sim.500-15.delly	0	–	–	4.8	0.2	–	–	–
venter-sim.500-15.gasv	4686	55.3	**0.1**	30.8	0.9	39.6	7.6	51.7
venter-sim.500-15.pindel	5336	94.5	0.0	60.4	3.9	73.7	**0.6**	**1.4**
venter-sim.500-15.svdetect	2	0.0	0.0	0.0	0.0	–	–	–
**Length Range 50–99** (1,784 true deletions)								
venter-sim.500-15.breakdancer	4979	13.7	0.1	3.1	1.2	5.1	36.4	49.1
venter-sim.500-15.clever	1157	93.9	0.0	**71.4**	**27.9**	**81.1**	9.3	25.7
venter-sim.500-15.delly	1	**100.0**	0.0	6.6	1.1	12.3	52.0	27.0
venter-sim.500-15.gasv	518	79.7	0.2	26.3	1.8	39.5	9.8	48.3
venter-sim.500-15.pindel	614	82.7	0.0	28.5	2.3	42.4	**3.7**	**3.4**
venter-sim.500-15.svdetect	7	0.0	**14.3**	0.0	0.0	–	–	–
**Length Range 100–249** (1,122 true deletions)								
venter-sim.500-15.breakdancer	875	18.5	0.0	14.1	2.2	16.0	15.1	43.2
venter-sim.500-15.clever	750	**88.7**	0.0	**57.5**	**8.4**	**69.8**	12.2	30.3
venter-sim.500-15.delly	5422	20.1	0.0	47.5	4.4	28.3	53.4	36.6
venter-sim.500-15.gasv	449	58.1	**0.2**	23.6	1.2	33.6	10.7	59.7
venter-sim.500-15.pindel	375	71.2	0.0	22.9	1.2	34.7	**7.0**	**4.6**
venter-sim.500-15.svdetect	53	3.8	0.0	0.2	0.0	0.3	77.0	32.5
**Length Range 250–999** (1,504 true deletions)								
venter-sim.500-15.breakdancer	1202	82.0	**0.0**	65.4	0.9	72.8	4.1	59.4
venter-sim.500-15.clever	1151	**89.4**	**0.0**	68.2	1.5	**77.3**	6.4	15.1

venter-sim.500-15.delly	5297	19.9	**0.0**	**69.5**	**2.6**	30.9	14.4	11.8
venter-sim.500-15.gasv	116460	0.6	**0.0**	45.0	1.3	1.1	8.3	69.3
venter-sim.500-15.pindel	920	88.6	**0.0**	54.2	0.7	67.2	**0.2**	**0.2**
venter-sim.500-15.svdetect	176	23.3	**0.0**	2.6	0.0	4.7	41.0	44.9
**Length Range 1000–50000** (299 true deletions)								
venter-sim.500-15.breakdancer	291	61.9	**0.0**	60.5	0.7	61.2	3.7	58.4
venter-sim.500-15.clever	210	**86.7**	**0.0**	61.5	1.0	**72.0**	5.5	13.8
venter-sim.500-15.delly	872	22.9	**0.0**	**67.6**	**2.7**	34.2	3.8	5.7
venter-sim.500-15.gasv	471	24.2	**0.0**	38.5	1.3	29.7	6.6	70.6
venter-sim.500-15.pindel	459	36.2	**0.0**	54.5	0.7	43.5	**0.0**	**1.4**
venter-sim.500-15.svdetect	83	38.6	**0.0**	11.0	0.0	17.2	58.8	30.2

2.3 Table Legend

- **Abs.:** *Absolute number* of predictions made in this length range
- **Prec.:** *Precision*, the percentage of predictions in that length range that match a true deletion/insertion.
- **Mix.:** Percentage of predictions that don't match a true insertion/deletion but a *mixed insertion/deletion event* of the same/similar effective length.
- **Rec.:** *Recall*, the percentage of true insertions/deletions in that length range that have been discovered.
- **Exc.:** *Exclusive calls*: percentage of true insertions/deletions that are *only* discovered by this tool.
- **F:** *F-Measure*: $2 \cdot \text{precision} \cdot \text{recall}/(\text{precision} + \text{recall})$. This integrates precision and recall into one statistic.
- **∆Len.:** *Length difference*: average length difference between prediction and true insertion/deletion (averaged over all predictions that match a true annotation)
- **Dist.:** *Distance*: average center distance between prediction and true insertion/deletion (averaged over all predictions that match a true annotation)
