# Supplementary material for: SV-AUTOPILOT: optimized, automated construction of structural variation discovery and benchmarking pipelines
Source: BMC Genomics. 2015 Mar 25;16(1):238. doi: 10.1186/s12864-015-1376-9 (PMC4520269; doi:10.1186/s12864-015-1376-9)
Supplement: Additional file 1: — The data sets supporting the results of this article are available in the as part of the SV-AUTOPILOT virtual machine, in https://bioimg.org/sv-autopilot . The scripts used as the basis for the virtual machine described in this article are available via the GitHub repository, in https://github.com/ALLBio/allbiotc2/. [file 12864_2015_1376_MOESM1_ESM.zip › 1993348534130930_add4.pdf]

1 Command line

```
../../allbiotc2/evaluation/evaluate-sv-predictions2 -R 20-49,50-99,100-249,250-999,1000-50000 -e
    human_sd15_o100z100 -o 100 -z 100 -L ../../data/reference_human/venter.phased.b37.chr21.nodots.vcf
    mean500-stddev15-cov30.breakdancer.vcf mean500-stddev15-cov30.clever.vcf mean500-stddev15-cov30.
    delly.vcf mean500-stddev15-cov30.gasv.vcf mean500-stddev15-cov30.pindel.vcf mean500-stddev15-cov30.
    prism.vcf mean500-stddev15-cov30.svdetect.vcf
```

2 Overall performance

2.1 Insertions

	Abs.	Prec.	Mix.	Rec.	Exc.	F.	ΔLen.	Dist.
**Length Range 20–49** (136 true insertions)								
m500-sd15-cov30.breakdancer	0	–	–	2.9	0.7	–	–	–
m500-sd15-cov30.clever	137	**88.3**	0.7	**72.8**	**25.0**	**79.8**	**5.7**	23.3
m500-sd15-cov30.delly	0	–	–	0.0	0.0	–	–	–
m500-sd15-cov30.gasv	0	–	–	0.0	0.0	–	–	–
m500-sd15-cov30.pindel	117	88.0	**0.9**	58.8	11.8	70.5	6.6	**7.6**
m500-sd15-cov30.prism	0	–	–	0.0	0.0	–	–	–
m500-sd15-cov30.svdetect	1	0.0	0.0	0.7	0.0	0.0	–	–
**Length Range 50–99** (37 true insertions)								
m500-sd15-cov30.breakdancer	62	8.1	0.0	0.0	0.0	0.0	37.8	48.6
m500-sd15-cov30.clever	35	80.0	**2.9**	**83.8**	**37.8**	**81.8**	7.6	36.6
m500-sd15-cov30.delly	0	–	–	0.0	0.0	–	–	–
m500-sd15-cov30.gasv	0	–	–	0.0	0.0	–	–	–
m500-sd15-cov30.pindel	14	**85.7**	0.0	45.9	2.7	59.8	**2.8**	**14.9**
m500-sd15-cov30.prism	0	–	–	0.0	0.0	–	–	–
m500-sd15-cov30.svdetect	4	50.0	0.0	2.7	0.0	5.1	25.5	33.5
**Length Range 100–249** (30 true insertions)								
m500-sd15-cov30.breakdancer	26	3.8	**0.0**	0.0	0.0	0.0	99.0	87.0
m500-sd15-cov30.clever	26	**88.5**	**0.0**	**60.0**	**53.3**	**71.5**	**18.2**	**37.8**
m500-sd15-cov30.delly	0	–	–	0.0	0.0	–	–	–
m500-sd15-cov30.gasv	0	–	–	0.0	0.0	–	–	–
m500-sd15-cov30.pindel	0	–	–	6.7	0.0	–	–	–
m500-sd15-cov30.prism	0	–	–	0.0	0.0	–	–	–
m500-sd15-cov30.svdetect	3	0.0	**0.0**	0.0	0.0	–	–	–
**Length Range 250–999** (19 true insertions)								
m500-sd15-cov30.breakdancer	5	0.0	**0.0**	0.0	0.0	–	–	–
m500-sd15-cov30.clever	1	**100.0**	**0.0**	**5.3**	**5.3**	**10.0**	**6.0**	**3.0**
m500-sd15-cov30.delly	0	–	–	0.0	0.0	–	–	–
m500-sd15-cov30.gasv	0	–	–	0.0	0.0	–	–	–
m500-sd15-cov30.pindel	0	–	–	0.0	0.0	–	–	–
m500-sd15-cov30.prism	0	–	–	0.0	0.0	–	–	–
m500-sd15-cov30.svdetect	0	–	–	0.0	0.0	–	–	–
**Length Range 1000–50000** (10 true insertions)								
m500-sd15-cov30.breakdancer	0	–	–	**0.0**	**0.0**	–	–	–
m500-sd15-cov30.clever	0	–	–	**0.0**	**0.0**	–	–	–
m500-sd15-cov30.delly	0	–	–	**0.0**	**0.0**	–	–	–
m500-sd15-cov30.gasv	0	–	–	**0.0**	**0.0**	–	–	–
m500-sd15-cov30.pindel	0	–	–	**0.0**	**0.0**	–	–	–
m500-sd15-cov30.prism	0	–	–	**0.0**	**0.0**	–	–	–
m500-sd15-cov30.svdetect	0	–	–	**0.0**	**0.0**	–	–	–

2.2 Deletions

	Abs.	Prec.	Mix.	Rec.	Exc.	F.	ΔLen.	Dist.
**Length Range 20–49** (118 true deletions)								
m500-sd15-cov30.breakdancer	0	–	–	8.5	0.0	–	–	–
m500-sd15-cov30.clever	111	92.8	0.9	83.1	3.4	**87.7**	7.9	21.8
m500-sd15-cov30.delly	0	–	–	5.9	0.0	–	–	–
m500-sd15-cov30.gasv	64	40.6	**3.1**	20.3	0.0	27.1	9.2	45.4
m500-sd15-cov30.pindel	82	90.2	0.0	62.7	0.0	74.0	**0.3**	**1.6**
m500-sd15-cov30.prism	270	48.5	0.4	**88.1**	**4.2**	62.6	4.0	9.9
m500-sd15-cov30.svdetect	12	**100.0**	0.0	4.2	0.0	8.1	39.6	26.4
**Length Range 50–99** (33 true deletions)								
m500-sd15-cov30.breakdancer	85	11.8	0.0	0.0	0.0	0.0	31.3	34.8
m500-sd15-cov30.clever	29	89.7	**3.4**	**90.9**	**6.1**	**90.3**	12.3	27.0
m500-sd15-cov30.delly	0	–	–	12.1	0.0	–	–	–
m500-sd15-cov30.gasv	11	72.7	0.0	27.3	0.0	39.7	**5.2**	50.2
m500-sd15-cov30.pindel	13	92.3	0.0	27.3	0.0	42.1	12.2	**5.0**
m500-sd15-cov30.prism	105	36.2	0.0	63.6	3.0	46.1	19.9	14.8
m500-sd15-cov30.svdetect	10	**100.0**	0.0	75.8	0.0	86.2	29.6	22.2
**Length Range 100–249** (19 true deletions)								
m500-sd15-cov30.breakdancer	14	35.7	0.0	26.3	5.3	30.3	3.0	35.1

m500-sd15-cov30.clever	13	**84.6**	0.0	**52.6**	**10.5**	**64.9**	14.7	36.7
m500-sd15-cov30.delly	79	20.3	1.3	36.8	0.0	26.1	59.7	42.4
m500-sd15-cov30.gasv	8	50.0	0.0	21.1	0.0	29.6	15.0	70.0
m500-sd15-cov30.pindel	7	42.9	0.0	26.3	0.0	32.6	**0.0**	**0.0**
m500-sd15-cov30.prism	16	31.2	**6.2**	36.8	**10.5**	33.8	24.0	15.6
m500-sd15-cov30.svdetect	17	64.7	0.0	47.4	0.0	54.7	53.7	27.8
**Length Range 250–999** (19 true deletions)								
m500-sd15-cov30.breakdancer	15	86.7	**0.0**	68.4	0.0	**76.5**	2.7	56.2
m500-sd15-cov30.clever	16	75.0	**0.0**	63.2	0.0	68.6	4.1	15.0
m500-sd15-cov30.delly	94	17.0	**0.0**	**73.7**	**5.3**	27.7	22.9	23.8
m500-sd15-cov30.gasv	2216	0.3	**0.0**	36.8	0.0	0.6	5.4	61.6
m500-sd15-cov30.pindel	9	**100.0**	**0.0**	47.4	0.0	64.3	**0.0**	**0.0**
m500-sd15-cov30.prism	36	38.9	**0.0**	68.4	**5.3**	49.6	4.6	1.5
m500-sd15-cov30.svdetect	19	63.2	**0.0**	63.2	0.0	63.2	51.6	38.4
**Length Range 1000–50000** (4 true deletions)								
m500-sd15-cov30.breakdancer	2	**100.0**	**0.0**	**50.0**	0.0	**66.7**	3.0	54.0
m500-sd15-cov30.clever	4	25.0	**0.0**	25.0	0.0	25.0	7.0	0.5
m500-sd15-cov30.delly	12	16.7	**0.0**	**50.0**	**25.0**	25.0	3.0	6.5
m500-sd15-cov30.gasv	4	25.0	**0.0**	25.0	0.0	25.0	2.0	20.0
m500-sd15-cov30.pindel	10	10.0	**0.0**	25.0	0.0	14.3	**0.0**	**0.0**
m500-sd15-cov30.prism	5	20.0	**0.0**	25.0	0.0	22.2	**0.0**	**0.0**
m500-sd15-cov30.svdetect	9	11.1	**0.0**	25.0	0.0	15.4	45.0	24.5

2.3 Table Legend

- **Abs.:** *Absolute number* of predictions made in this length range
- **Prec.:** *Precision*, the percentage of predictions in that length range that match a true deletion/insertion.
- **Mix.:** Percentage of predictions that don't match a true insertion/deletion but a *mixed insertion/deletion event* of the same/similar effective length.
- **Rec.:** *Recall*, the percentage of true insertions/deletions in that length range that have been discovered.
- **Exc.:** *Exclusive calls*: percentage of true insertions/deletions that are *only* discovered by this tool.
- **F:** *F-Measure*: $2 \cdot \text{precision} \cdot \text{recall}/(\text{precision} + \text{recall})$. This integrates precision and recall into one statistic.
- **ΔLen.:** *Length difference*: average length difference between prediction and true insertion/deletion (averaged over all predictions that match a true annotation)
- **Dist.:** *Distance*: average center distance between prediction and true insertion/deletion (averaged over all predictions that match a true annotation)
