# Supplementary material for: SV-AUTOPILOT: optimized, automated construction of structural variation discovery and benchmarking pipelines
Source: BMC Genomics. 2015 Mar 25;16(1):238. doi: 10.1186/s12864-015-1376-9 (PMC4520269; doi:10.1186/s12864-015-1376-9)
Supplement: Additional file 1: — The data sets supporting the results of this article are available in the as part of the SV-AUTOPILOT virtual machine, in https://bioimg.org/sv-autopilot . The scripts used as the basis for the virtual machine described in this article are available via the GitHub repository, in https://github.com/ALLBio/allbiotc2/. [file 12864_2015_1376_MOESM1_ESM.zip › 1993348534130930_add7.pdf]

1 Command line

```
../../allbiotc2/evaluation/evaluate-sv-predictions2 -R 20-49,50-99,100-249,250-999,1000-50000 -e
    human_sd50_o50z20 -o 50 -z 20 -L ../../data/reference_human/venter.phased.b37.chr21.nodots.vcf
    mean500-stddev50-cov30.breakdancer.vcf mean500-stddev50-cov30.clever.vcf mean500-stddev50-cov30.
    delly.vcf mean500-stddev50-cov30.gasv.vcf mean500-stddev50-cov30.pindel.vcf mean500-stddev50-cov30.
    prism.vcf mean500-stddev50-cov30.svdetect.vcf
```

2 Overall performance

2.1 Insertions

	Abs.	Prec.	Mix.	Rec.	Exc.	F.	ΔLen.	Dist.
**Length Range 20–49** (136 true insertions)								
m500-sd50-cov30.breakdancer	0	–	–	0.0	0.0	–	–	–
m500-sd50-cov30.clever	0	–	–	0.0	0.0	–	–	–
m500-sd50-cov30.delly	0	–	–	0.0	0.0	–	–	–
m500-sd50-cov30.gasv	0	–	–	0.0	0.0	–	–	–
m500-sd50-cov30.pindel	132	**75.0**	**0.0**	**61.0**	**60.3**	**67.3**	**1.9**	**6.2**
m500-sd50-cov30.prism	0	–	–	0.0	0.0	–	–	–
m500-sd50-cov30.svdetect	11	9.1	**0.0**	0.7	0.0	1.4	8.0	37.0
**Length Range 50–99** (37 true insertions)								
m500-sd50-cov30.breakdancer	0	–	–	0.0	0.0	–	–	–
m500-sd50-cov30.clever	18	16.7	**0.0**	8.1	8.1	10.9	12.7	15.3
m500-sd50-cov30.delly	0	–	–	0.0	0.0	–	–	–
m500-sd50-cov30.gasv	0	–	–	0.0	0.0	–	–	–
m500-sd50-cov30.pindel	17	**52.9**	**0.0**	**18.9**	**18.9**	**27.9**	**0.0**	**7.1**
m500-sd50-cov30.prism	0	–	–	0.0	0.0	–	–	–
m500-sd50-cov30.svdetect	29	3.4	**0.0**	2.7	2.7	3.0	2.0	27.0
**Length Range 100–249** (30 true insertions)								
m500-sd50-cov30.breakdancer	23	0.0	**0.0**	0.0	0.0	–	–	–
m500-sd50-cov30.clever	42	**16.7**	**0.0**	**23.3**	**23.3**	**19.4**	**11.3**	**14.7**
m500-sd50-cov30.delly	0	–	–	0.0	0.0	–	–	–
m500-sd50-cov30.gasv	0	–	–	0.0	0.0	–	–	–
m500-sd50-cov30.pindel	0	–	–	0.0	0.0	–	–	–
m500-sd50-cov30.prism	0	–	–	0.0	0.0	–	–	–
m500-sd50-cov30.svdetect	42	0.0	**0.0**	0.0	0.0	–	–	–
**Length Range 250–999** (19 true insertions)								
m500-sd50-cov30.breakdancer	8	**0.0**	**0.0**	**0.0**	**0.0**	–	–	–
m500-sd50-cov30.clever	0	–	–	**0.0**	**0.0**	–	–	–
m500-sd50-cov30.delly	0	–	–	**0.0**	**0.0**	–	–	–
m500-sd50-cov30.gasv	0	–	–	**0.0**	**0.0**	–	–	–
m500-sd50-cov30.pindel	0	–	–	**0.0**	**0.0**	–	–	–
m500-sd50-cov30.prism	0	–	–	**0.0**	**0.0**	–	–	–
m500-sd50-cov30.svdetect	0	–	–	**0.0**	**0.0**	–	–	–
**Length Range 1000–50000** (10 true insertions)								
m500-sd50-cov30.breakdancer	0	–	–	**0.0**	**0.0**	–	–	–
m500-sd50-cov30.clever	0	–	–	**0.0**	**0.0**	–	–	–
m500-sd50-cov30.delly	0	–	–	**0.0**	**0.0**	–	–	–
m500-sd50-cov30.gasv	0	–	–	**0.0**	**0.0**	–	–	–
m500-sd50-cov30.pindel	0	–	–	**0.0**	**0.0**	–	–	–
m500-sd50-cov30.prism	0	–	–	**0.0**	**0.0**	–	–	–
m500-sd50-cov30.svdetect	0	–	–	**0.0**	**0.0**	–	–	–

2.2 Deletions

	Abs.	Prec.	Mix.	Rec.	Exc.	F.	ΔLen.	Dist.
**Length Range 20–49** (118 true deletions)								
m500-sd50-cov30.breakdancer	0	–	–	0.0	0.0	–	–	–
m500-sd50-cov30.clever	11	27.3	0.0	1.7	0.0	3.2	8.0	14.3
m500-sd50-cov30.delly	0	–	–	0.0	0.0	–	–	–
m500-sd50-cov30.gasv	461	1.1	0.0	3.4	0.0	1.6	16.0	33.0
m500-sd50-cov30.pindel	74	**87.8**	0.0	54.2	0.0	**67.1**	**0.2**	**1.1**
m500-sd50-cov30.prism	234	47.9	**0.9**	**83.9**	**28.8**	61.0	1.3	3.0
m500-sd50-cov30.svdetect	31	0.0	0.0	0.0	0.0	–	–	–
**Length Range 50–99** (33 true deletions)								
m500-sd50-cov30.breakdancer	0	–	–	0.0	0.0	–	–	–
m500-sd50-cov30.clever	16	56.2	0.0	30.3	0.0	39.4	8.6	17.7
m500-sd50-cov30.delly	0	–	–	0.0	0.0	–	–	–
m500-sd50-cov30.gasv	112	1.8	0.0	3.0	0.0	2.2	17.0	32.5
m500-sd50-cov30.pindel	10	**90.0**	0.0	27.3	0.0	**41.9**	**0.0**	**1.7**
m500-sd50-cov30.prism	115	22.6	0.9	**66.7**	**21.2**	33.8	1.9	3.8
m500-sd50-cov30.svdetect	58	3.4	**1.7**	6.1	3.0	4.4	9.5	23.2
**Length Range 100–249** (19 true deletions)								
m500-sd50-cov30.breakdancer	16	6.2	0.0	5.3	0.0	5.7	16.0	49.0

m500-sd50-cov30.clever	22	31.8	**4.5**	**36.8**	**21.1**	**34.1**	11.0	15.2
m500-sd50-cov30.delly	51	5.9	0.0	15.8	0.0	8.6	2.7	14.0
m500-sd50-cov30.gasv	12	0.0	0.0	0.0	0.0	–	–	–
m500-sd50-cov30.pindel	6	**50.0**	0.0	15.8	0.0	24.0	**0.0**	**0.0**
m500-sd50-cov30.prism	147	4.8	0.7	26.3	5.3	8.1	**0.0**	3.7
m500-sd50-cov30.svdetect	171	0.6	0.6	5.3	0.0	1.1	5.0	40.5
**Length Range 250–999** (19 true deletions)								
m500-sd50-cov30.breakdancer	18	11.1	**0.0**	10.5	0.0	10.8	5.5	21.8
m500-sd50-cov30.clever	17	64.7	**0.0**	**57.9**	0.0	**61.1**	8.5	8.9
m500-sd50-cov30.delly	83	1.2	**0.0**	5.3	0.0	2.0	11.0	5.5
m500-sd50-cov30.gasv	3317	0.1	**0.0**	10.5	**10.5**	0.1	2.5	25.2
m500-sd50-cov30.pindel	11	**81.8**	**0.0**	47.4	0.0	60.0	**0.0**	**0.0**
m500-sd50-cov30.prism	40	35.0	**0.0**	**57.9**	5.3	43.6	1.3	3.1
m500-sd50-cov30.svdetect	240	0.4	**0.0**	5.3	0.0	0.8	8.0	6.0
**Length Range 1000–50000** (4 true deletions)								
m500-sd50-cov30.breakdancer	2	0.0	**0.0**	0.0	0.0	–	–	–
m500-sd50-cov30.clever	3	**33.3**	**0.0**	25.0	0.0	**28.6**	1.0	0.5
m500-sd50-cov30.delly	10	10.0	**0.0**	25.0	0.0	14.3	**0.0**	1.0
m500-sd50-cov30.gasv	5	0.0	**0.0**	0.0	0.0	–	–	–
m500-sd50-cov30.pindel	12	16.7	**0.0**	**50.0**	**25.0**	25.0	**0.0**	**0.0**
m500-sd50-cov30.prism	6	16.7	**0.0**	25.0	0.0	20.0	**0.0**	**0.0**
m500-sd50-cov30.svdetect	6	0.0	**0.0**	0.0	0.0	–	–	–

2.3 Table Legend

- **Abs.:** *Absolute number* of predictions made in this length range
- **Prec.:** *Precision*, the percentage of predictions in that length range that match a true deletion/insertion.
- **Mix.:** Percentage of predictions that don't match a true insertion/deletion but a *mixed insertion/deletion event* of the same/similar effective length.
- **Rec.:** *Recall*, the percentage of true insertions/deletions in that length range that have been discovered.
- **Exc.:** *Exclusive calls*: percentage of true insertions/deletions that are *only* discovered by this tool.
- **F:** *F-Measure*: $2 \cdot \text{precision} \cdot \text{recall}/(\text{precision} + \text{recall})$. This integrates precision and recall into one statistic.
- **ΔLen.:** *Length difference*: average length difference between prediction and true insertion/deletion (averaged over all predictions that match a true annotation)
- **Dist.:** *Distance*: average center distance between prediction and true insertion/deletion (averaged over all predictions that match a true annotation)
